# Supplementary material for: Pathologist-like explainable AI for interpretable Gleason grading in prostate cancer
Source: Nat Commun. 2025 Oct 8;16:8959. doi: 10.1038/s41467-025-64712-4 (PMC12508442; doi:10.1038/s41467-025-64712-4)
Supplement: Supplementary file 1 — Supplementary Information [file 41467_2025_64712_MOESM1_ESM.docx]

Pathologist-like explainable AI for interpretable Gleason grading in prostate cancer

# Supplementary Tables

## Initial Ontology

**Supplementary Table 1:** **Initial Ontology for Gleason Pattern 3.** Explanatory Ontology translated back from the German Ontology, with the numbering used in result figures on the right.

| Gleason Pattern | Feature | Number |
| --- | --- | --- |
| 3 | single, individual atypical glands separated from each other | 3.01 |
| 3 | atypical glands with an irregularly separated, ragged, poorly defined edge | 3.02 |
| 3 | atypical glands are looser than a nodule and are infiltrative | 3.03 |
| 3 | either minute or large and cyst-like atrophic atypical glands | 3.04 |
| 3 | atypical glands lying very closely together (with little stroma between adjacent atypical glands) | 3.05 |
| 3 | well-formed, relatively uniform atypical glands with evenly distributed lumina | 3.06 |
| 3 | compressed or angular atypical glands | 3.07 |
| 3 | atypical glands infiltrate between benign glands | 3.08 |

**Supplementary Table 2:** **Initial Ontology for Gleason Pattern 4.** Explanatory Ontology translated back from the German Ontology, with the numbering used in result figures on the right.

| Gleason Pattern | Feature | Number |
| --- | --- | --- |
| 4 | slit-like lumina | 4.01 |
| 4 | large atypical glands | 4.02 |
| 4 | irregular contours, jagged edges of atypical glands | 4.03 |
| 4 | atypical glands fused or grown together into cords or chains | 4.04 |
| 4 | irregular distribution of lumina | 4.05 |
| 4 | atypical glands very close together (with little or no stroma) | 4.06 |
| 4 | Cribriform | 4.07 |
| 4 | Cribriform: larger than a normal prostate gland; tends to fragmentation | 4.08 |
| 4 | Cribriform: confluent sheet of contiguous carcinoma cells with multiple glandular lumina that are easily visible at low power (objective magnification 10x) | 4.09 |
| 4 | Cribriform: single or fused glandular structures connected to each other (no intervening stroma or mucin) | 4.10 |
| 4 | Hypernephroid pattern | 4.11 |
| 4 | Hypernephroid pattern: nests of clear cells resembling renal cell carcinoma | 4.12 |
| 4 | Hypernephroid pattern: small, hyperchromatic nuclei | 4.13 |
| 4 | Hypernephroid pattern: fusion of acini into more solid sheets with the appearance of back-to-back glands without intervening stroma | 4.14 |
| 4 | Glomeruloid pattern | 4.15 |
| 4 | Glomeruloid pattern: rare small cribriform variant resembling glomerulus structures of kidney | 4.16 |
| 4 | Glomeruloid pattern: contains a tuft of cells that is largely detached from its surrounding duct space except for a single point of attachment | 4.17 |

**Supplementary Table 3:** **Initial Ontology for Gleason Pattern 5.** Explanatory Ontology translated back from the German Ontology, with the numbering used in result figures on the right.

| Gleason Pattern | Feature | Number |
| --- | --- | --- |
| 5 | solid tumor cell clusters with nonpolar nuclei around a lumen | 5.01 |
| 5 | Presence of definite comedonecrosis (central necrosis) | 5.02 |
| 5 | Presence of definite comedonecrosis (central necrosis): with intraluminal necrotic cells | 5.03 |
| 5 | Presence of definite comedonecrosis (central necrosis): with karyorrhexis within papillary, cribriform spaces | 5.04 |
| 5 | Single cells | 5.05 |
| 5 | Single cells: forming cords | 5.06 |
| 5 | Single cells: with vacuoles (signet ring cells) lacking glandular lumina | 5.07 |

## Annotator Agreement for Sub-Explanations


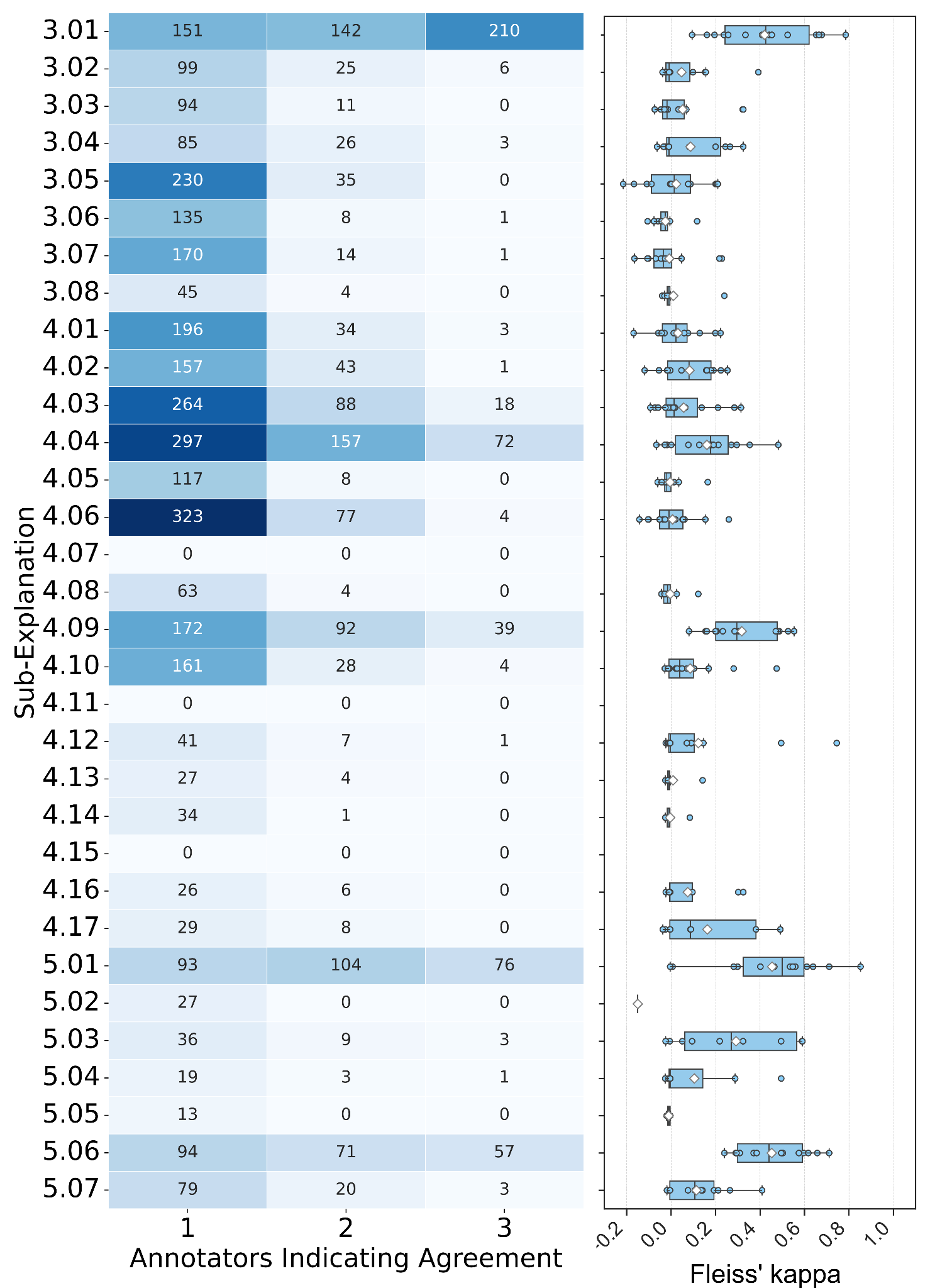


**Supplementary Fig. 1: Agreement of annotators for explanations on the image-level.** Heatmap containing the number of TMA core images in which n out of the three annotators indicated the presence of the sub-explanations and the resulting Fleiss’ kappa for groups of three raters (on the right). In the box plot, dots represent the groups of *n*=3 annotators, diamonds represent the mean, boxes represent quartiles and the centre line the median, and whiskers extend to the maxima and minima within 1.5 of the inter-quartile range. As not all groups used all categories, the number of groups per category varies, and can be derived from Supplementary Table 9 and Supplementary Table 10. Source data are provided as a Source Data file.

## Fleiss’ Kappa Tables

**Supplementary Table 4:** **Fleiss’ Kappa for patterns and explanations.** Fleiss’ kappa (κ) and 95% confidence interval (bootstrap: 10.000 resamples) within each label for Gleason patterns and explanations.

| Label | κ | 95% CI |
| --- | --- | --- |
| 3 | 0.784 | [0.751, 0.813] |
| 4 | 0.684 | [0.647, 0.719] |
| 5 | 0.786 | [0.749, 0.819] |
| 3 - compressed glands | 0.145 | [0.096, 0.203] |
| 3 - individual glands | 0.710 | [0.676, 0.744] |
| 4 - cribriform glands | 0.431 | [0.384, 0.479] |
| 4 - glomeruloid glands | 0.329 | [0.203, 0.480] |
| 4 - poorly formed glands | 0.532 | [0.493, 0.571] |
| 5 - comedonecrosis | 0.347 | [0.229, 0.476] |
| 5 - cords | 0.532 | [0.475, 0.590] |
| 5 - groups of tumor cells | 0.549 | [0.500, 0.598] |
| 5 - single cells | 0.180 | [0.114, 0.270] |

**Supplementary Table 5: Fleiss’ kappa for sub-explanations 3.01 to 4.08.** Fleiss’ kappa and 95% interval (bootstrap: 10.000 resamples) within each label for sub-explanations 3.01 to 4.08. If no value is given, the label didn’t occur according to all raters.

| Sub-Explanation | κ | 95% CI |
| --- | --- | --- |
| 3.01 | 0.577 | [0.535, 0.616] |
| 3.02 | 0.214 | [0.146, 0.304] |
| 3.03 | 0.059 | [0.016, 0.114] |
| 3.04 | 0.201 | [0.139, 0.284] |
| 3.05 | 0.020 | [-0.11, 0.056] |
| 3.06 | 0.022 | [-0.014, 0.089] |
| 3.07 | 0.020 | [-0.015, 0.074] |
| 3.08 | 0.059 | [0.006, 0.146] |
| 4.01 | 0.075 | [0.033, 0.126] |
| 4.02 | 0.116 | [0.076, 0.163] |
| 4.03 | 0.149 | [0.104, 0.200] |
| 4.04 | 0.246 | [0.202, 0.292] |
| 4.05 | 0.017 | [-0.014, 0.065] |
| 4.06 | 0.026 | [-0.008, 0.063] |
| 4.07 | - | [-, -] |
| 4.08 | 0.034 | [-0.006, 0.102] |

**Supplementary Table 6: Fleiss’ kappa for sub-explanations 4.09 to 5.07.** Fleiss’ kappa and 95% interval (bootstrap: 10.000 resamples) within each label for sub-explanations 4.09 to 5.07. If no value is given, the label didn’t occur according to all raters.

| Sub-Explanation | κ | 95% CI |
| --- | --- | --- |
| 4.09 | 0.339 | [0.288, 0.397] |
| 4.10 | 0.108 | [0.058, 0.173] |
| 4.11 | - | [-, -] |
| 4.12 | 0.156 | [0.070, 0.303] |
| 4.13 | 0.104 | [0.025, 0.221] |
| 4.14 | 0.016 | [-0.012, 0.129] |
| 4.15 | - | [-, -] |
| 4.16 | 0.147 | [0.061, 0.259] |
| 4.17 | 0.165 | [0.080, 0.262] |
| 5.01 | 0.549 | [0.500, 0.600] |
| 5.02 | -0.009 | [-0.013, -0.006] |
| 5.03 | 0.271 | [0.155, 0.431] |
| 5.04 | 0.207 | [0.063, 0.495] |
| 5.05 | -0.004 | [-0.007, -0.002] |
| 5.06 | 0.532 | [0.473, 0.589] |
| 5.07 | 0.193 | [0.123, 0.286] |

**Supplementary Table 7:** **Fleiss’ Kappa within groups.** Fleiss’ kappa (κ) and 95% confidence interval (bootstrap: 10.000 resamples) within each group of raters for Gleason patterns (GP), explanations (expl.), and sub-explanations (sub-expl.).

| Group | κ (GP) | 95% CI (GP) | κ (expl.) | 95% CI (expl.) | κ (sub-expl.) | 95% CI (sub-expl.) |
| --- | --- | --- | --- | --- | --- | --- |
| 1.1 | 0.532 | [0.443, 0.620] | 0.357 | [0.294, 0.425] | 0.220 | [0.170, 0.281] |
| 1.2 | 0.837 | [0.796, 0.896] | 0.698 | [0.632, 0.756] | 0.295 | [0.241, 0.353] |
| 1.3 | 0.695 | [0.605, 0.774] | 0.461 | [0.396, 0.532] | 0.249 | [0.197, 0.308] |
| 2.1 | 0.729 | [0.652, 0.805] | 0.496 | [0.436, 0.556] | 0.355 | [0.310, 0.401] |
| 2.2 | 0.854 | [0.794, 0.907] | 0.681 | [0.621, 0.737] | 0.362 | [0.313, 0.415] |
| 2.3 | 0.829 | [0.763, 0.882] | 0.577 | [0.522, 0.632] | 0.388 | [0.338, 0.440] |
| 3.1 | 0.720 | [0.651, 0.786] | 0.522 | [0.463, 0.580] | 0.125 | [0.094, 0.162] |
| 3.2 | 0.779 | [0.703, 0.839] | 0.646 | [0.581, 0.708] | 0.343 | [0.283, 0.408] |
| 3.3 | 0.779 | [0.703, 0.837] | 0.663 | [0.599, 0.722] | 0.455 | [0.394, 0.516] |
| 3.4 | 0.756 | [0.686, 0.817] | 0.598 | [0.538, 0.658] | 0.374 | [0.323, 0.427] |
| 3.5 | 0.764 | [0.697, 0.822] | 0.663 | [0.601, 0.719] | 0.419 | [0.361, 0.477] |
| 3.6 | 0.681 | [0.606, 0.751] | 0.519 | [0.457, 0.581] | 0.318 | [0.265, 0.374] |
| 19.1 | 0.919 | [0.863, 0.954] | 0.638 | [0.580, 0.696] | 0.228 | [0.185, 0.276] |
| 19.2 | 0.935 | [0.888, 0.965] | 0.855 | [0.807, 0.895] | 0.388 | [0.337, 0.443] |

| Label | Group 1.1 | Group 1.2 | Group 1.3 | Group 2.1 | Group 2.2 | Group 2.3 | Group 3.1 | Group 3.2 | Group 3.3 | Group 3.4 | Group 3.5 | Group 3.6 | Group 19.1 | Group 19.2 |
| --- | --- | --- | --- | --- | --- | --- | --- | --- | --- | --- | --- | --- | --- | --- |
| Gleason Pattern | | | | | | | | | | | | | | |
| 3 | 0.273 | 0.799 | 0.592 | 0.735 | 0.800 | 0.397 | 0.664 | 0.729 | 0.843 | 0.582 | 0.807 | 0.826 | 0.920 | 0.854 |
| 4 | 0.225 | 0.666 | 0.516 | 0.633 | 0.801 | 0.711 | 0.560 | 0.763 | 0.699 | 0.697 | 0.578 | 0.557 | 0.759 | 0.937 |
| 5 | 0.457 | 0.861 | 0.689 | 0.676 | 0.788 | 0.909 | 0.845 | 0.804 | 0.727 | 0.886 | 0.636 | 0.644 | 1.00 | 1.00 |
| Explanation | | | | | | | | | | | | | | |
| 3 - compressed glands | -0.005 | 0.091 | -0.043 | 0.277 | 0.100 | -0.009 | 0.102 | -0.006 | -0.103 | -0.109 | -0.130 | 0.210 | 0.399 | -0.036 |
| 3 - individual glands | 0.237 | 0.755 | 0.460 | 0.735 | 0.721 | 0.288 | 0.549 | 0.652 | 0.824 | 0.582 | 0.807 | 0.664 | 0.418 | 0.854 |
| 4 - cribriform glands | 0.400 | 0.611 | 0.347 | 0.536 | 0.244 | 0.559 | 0.213 | 0.422 | 0.606 | 0.243 | 0.404 | 0.261 | 0.280 | 0.463 |
| 4 - glomeruloid glands | -0.005 | -0.011 | 0.531 | 0.067 | -0.031 | 0.237 | 0.198 | -0.005 | -0.005 | NA | -0.008 | 0.796 | 0.457 | 0.658 |
| 4 - poorly formed glands | -0.010 | 0.412 | 0.201 | 0.528 | 0.699 | 0.301 | 0.486 | 0.594 | 0.661 | 0.624 | 0.519 | 0.437 | 0.690 | 0.858 |
| 5 - comedonecrosis | -0.053 | 0.655 | -0.006 | 0.152 | 0.495 | 0.618 | 0.096 | 0.590 | 0.852 | NA | -0.004 | 0.076 | NA | NA |
| 5 - cords | 0.309 | 0.503 | 0.596 | 0.293 | 0.575 | 0.711 | 0.291 | 0.658 | 0.240 | 0.617 | 0.294 | 0.371 | 0.385 | 0.496 |
| 5 - groups of tumor cells | 0.299 | 0.464 | 0.006 | 0.281 | 0.535 | 0.458 | 0.610 | 0.711 | 0.559 | 0.402 | 0.547 | 0.638 | 0.852 | -0.004 |
| 5 - single cells | 0.264 | -0.006 | 0.193 | 0.091 | 0.069 | 0.212 | -0.016 | 0.136 | 0.304 | -0.012 | -0.019 | 0.060 | 0.235 | NA |

**Supplementary Table 8:** **Fleiss’ kappa per group and label for Gleason patterns and** **explanations.** Missing values (NA) are due to explanations not used by the annotators in the group’s data set.

**Supplementary Table 9:** **Fleiss’ kappa per group and label for** **sub-explanations 3.01 to 4.08.** Missing values (NA) are due to explanations not used by the annotators in the group’s data set (part 1).

| Sub-Explanation | Group 1.1 | Group 1.2 | Group 1.3 | Group 2.1 | Group 2.2 | Group 2.3 | Group 3.1 | Group 3.2 | Group 3.3 | Group 3.4 | Group 3.5 | Group 3.6 | Group 19.1 | Group 19.2 |
| --- | --- | --- | --- | --- | --- | --- | --- | --- | --- | --- | --- | --- | --- | --- |
| 3.01 | 0.237 | 0.409 | 0.161 | 0.653 | 0.422 | 0.094 | 0.195 | 0.524 | 0.678 | 0.453 | 0.785 | 0.334 | 0.257 | 0.665 |
| 3.02 | -0.005 | 0.153 | -0.030 | 0.156 | -0.020 | -0.009 | 0.042 | -0.030 | -0.019 | -0.024 | 0.039 | 0.098 | 0.392 | -0.009 |
| 3.03 | NA | 0.322 | NA | NA | -0.042 | 0.325 | -0.074 | -0.015 | NA | -0.046 | -0.023 | -0.030 | 0.033 | 0.068 |
| 3.04 | NA | 0.200 | NA | -0.027 | 0.244 | NA | 0.081 | -0.010 | -0.034 | 0.265 | -0.063 | -0.013 | -0.010 | 0.324 |
| 3.05 | NA | 0.087 | 0.014 | 0.200 | 0.205 | -0.004 | -0.215 | 0.087 | -0.109 | 0.077 | -0.167 | 0.210 | -0.088 | 0.002 |
| 3.06 | NA | -0.060 | -0.006 | NA | -0.031 | 0.117 | -0.106 | -0.020 | -0.024 | -0.077 | -0.042 | -0.005 | -0.026 | -0.027 |
| 3.07 | NA | -0.041 | -0.012 | 0.229 | -0.069 | NA | 0.047 | -0.046 | -0.103 | -0.165 | -0.107 | 0.217 | -0.020 | -0.027 |
| 3.08 | NA | -0.006 | -0.006 | NA | -0.005 | NA | -0.040 | -0.030 | NA | 0.240 | -0.015 | -0.004 | -0.015 | -0.018 |
| 4.01 | -0.046 | -0.058 | 0.014 | 0.076 | 0.031 | 0.199 | 0.012 | -0.030 | 0.029 | 0.222 | -0.043 | 0.129 | -0.169 | 0.059 |
| 4.02 | -0.119 | -0.055 | -0.018 | 0.190 | 0.047 | 0.158 | -0.004 | 0.179 | NA | -0.016 | 0.081 | 0.244 | 0.161 | 0.254 |
| 4.03 | -0.073 | 0.137 | 0.017 | 0.284 | -0.093 | -0.002 | 0.017 | 0.010 | -0.014 | 0.314 | 0.064 | -0.058 | -0.026 | 0.211 |
| 4.04 | -0.019 | 0.182 | -0.066 | 0.173 | 0.353 | -0.029 | 0.077 | 0.127 | 0.482 | 0.213 | 0.191 | 0.271 | 0.295 | 0.001 |
| 4.05 | -0.020 | -0.029 | -0.012 | -0.061 | 0.013 | 0.034 | -0.042 | -0.020 | -0.019 | -0.030 | -0.043 | 0.165 | -0.005 | 0.001 |
| 4.06 | -0.101 | -0.052 | -0.104 | -0.143 | 0.154 | 0.034 | 0-019 | -0.027 | 0.051 | 0.060 | 0.260 | 0.007 | 0.054 | -0.052 |
| 4.07 | NA | NA | NA | NA | NA | NA | NA | NA | NA | NA | NA | NA | NA | NA |
| 4.08 | -0.043 | -0.035 | -0.024 | -0.044 | -0.005 | 0.122 | -0.004 | -0.030 | NA | NA | NA | -0.009 | 0.025 | NA |

**Supplementary Table 10:** **Fleiss’ kappa per group and label for sub-explanations 4.09 to 5.07.** Missing values (NA) are due to explanations not used by the annotators in the group’s data set (part 2).

| Sub-Explanation | Group 1.1 | Group 1.2 | Group 1.3 | Group 2.1 | Group 2.2 | Group 2.3 | Group 3.1 | Group 3.2 | Group 3.3 | Group 3.4 | Group 3.5 | Group 3.6 | Group 19.1 | Group 19.2 |
| --- | --- | --- | --- | --- | --- | --- | --- | --- | --- | --- | --- | --- | --- | --- |
| 4.09 | 0.232 | 0.526 | 0.487 | 0.553 | 0.205 | 0.305 | 0.155 | 0.311 | 0.480 | 0.080 | 0.286 | 0.199 | 0.161 | 0.471 |
| 4.10 | -0.025 | 0.091 | 0.103 | 0.281 | -0.028 | -0.011 | 0.022 | 0.027 | 0.072 | 0.475 | 0.169 | 0.050 | -0.007 | -0.013 |
| 4.11 | NA | NA | NA | NA | NA | NA | NA | NA | NA | NA | NA | NA | NA | NA |
| 4.12 | -0.005 | NA | -0.024 | 0.091 | 0.745 | 0.145 | -0.016 | 0.071 | 0.495 | -0.004 | NA | -0.013 | -0.010 | -0.004 |
| 4.13 | -0.010 | NA | NA | 0.142 | -0.026 | -0.013 | -0.004 | -0.015 | NA | NA | NA | NA | -0.015 | NA |
| 4.14 | NA | 0.084 | -0.018 | -0.005 | -0.005 | -0.013 | -0.016 | -0.010 | NA | -0.004 | -0.008 | -0.026 | -0.026 | NA |
| 4.15 | NA | NA | NA | NA | NA | NA | NA | NA | NA | NA | NA | NA | NA | NA |
| 4.16 | -0.005 | -0.006 | -0.024 | -0.011 | -0.005 | 0.325 | 0.096 | -0.005 | -0.005 | NA | -0.008 | -0.009 | 0.302 | 0.324 |
| 4.17 | NA | -0.006 | 0.382 | 0.087 | -0.026 | -0.004 | -0.038 | NA | NA | NA | NA | 0.491 | 0.089 | 0.491 |
| 5.01 | 0.299 | 0.464 | 0.007 | 0.281 | 0.535 | 0.458 | 0.610 | 0.711 | 0.559 | 0.402 | 0.547 | 0.638 | 0.852 | -0.004 |
| 5.02 | -0.150 | NA | NA | NA | NA | NA | NA | NA | NA | NA | NA | NA | NA | NA |
| 5.03 | 0.324 | 0.589 | -0.006 | 0.051 | 0.495 | 0.218 | -0.025 | 0.590 | 0.590 | NA | NA | 0.094 | NA | NA |
| 5.04 | NA | -0.006 | NA | -0.027 | NA | 0.288 | -0.012 | NA | 0.495 | NA | -0.004 | -0.004 | NA | NA |
| 5.05 | NA | NA | NA | -0.005 | -0.020 | NA | NA | NA | -0.014 | -0.004 | NA | -0.004 | -0.015 | NA |
| 5.06 | 0.309 | 0.503 | 0.596 | 0.293 | 0.575 | 0.711 | 0.291 | 0.658 | 0.240 | 0.617 | 0.294 | 0.371 | 0.385 | 0.496 |
| 5.07 | 0.264 | -0.006 | 0.193 | 0.106 | 0.141 | 0.212 | -0.016 | 0.136 | 0.409 | -0.012 | 0.019 | 0.076 | -0.005 | NA |

# Supplementary Notes

## Additional Results

### Ablation study: Tree Loss

As the ontology has a natural hierarchical structure, we experimented with using a hierarchical tree-structured loss function for our soft labels that optimized a loss function for all levels of our ontology simultaneously. The models predictions on the lower levels of the ontology (explanations or sub-explanations), were additionally remapped to higher levels, thereby jointly optimizing the (sub-)explanation and Gleason pattern segmentations.

Let $y \in\mathbb{R}^{C}$be a label distribution, with $y_{i}$ belonging to the i’th explanation or sub-explanation. Let $\phi(i)$ be the index of the corresponding class in the next higher ontology level (e.g. Gleason patterns for the explanations) belonging to the i’th explanation.

Given a loss-function $L$, we minimize the following loss-function for the prediction vector $p^{\mathsf{seg}},y^{\mathsf{seg}}\in\Delta(N)$, with $\Delta(N)$being the N-dimensional probability simplex:

(2) $\mathsf{Treeloss}_{L}(p^{\mathsf{seg}}, y^{\mathsf{seg}}) =\lambda L(p^{\mathsf{seg}}, y^{\mathsf{seg}}) +(1-\lambda) L(p^{\mathsf{map}},y^{\mathsf{map}} )$

, where ${p_{k}}^{\mathsf{map}} = \sum_{i, \phi(i) =k} p_{i}$ being the sum of (sub-)explanation probabilities that belong to k’th Gleason pattern or explanation and $y^{\mathsf{map}}$ defined analogously. We experimented with different values for $\lambda$ and set it to $\lambda=0.5$. When training on sub-explanations the remapping is performed twice, first to the explanations and then to the Gleason patterns.

As we did not notice significant improvements using this method, we did not include it into our main results, however some interesting observations can be found in Supplementary Fig. 2

### Results on the sub-explanations

As can be seen in Supplementary Fig. 2, when training on the sub-explanations, the quality of the segmentations dropped significantly, with the Macro Dice dropping below 0.11, while the Dice score remained at 0.67, even better than the best performing method for the explanations.

Our explanations for this behavior are the significant imbalance and inter-pathologist disagreements (see [Figure 2](https://docs.google.com/document/d/12SpMrcdW3LseAYLAlLt8UxaH2f4-exnavcTm3WOZLWQ/edit?pli=1#fig_imagecount) and Supplementary Fig. 1) that were present in the sub-explanations.

Additionally, when evaluating the SoftDiceLoss and soft label cross-entropy loss on the Gleason patterns, a dramatic decrease in segmentation performance could be observed, which is unacceptable for clinical practice.

However, utilizing a hierarchical loss through our TreeLoss (see [Ablation study: Tree Loss](https://docs.google.com/document/d/12SpMrcdW3LseAYLAlLt8UxaH2f4-exnavcTm3WOZLWQ/edit?pli=1#heading=h.u0jfy12jxzsi)) that trains on all three levels of the ontology hierarchy at the same time, we are able to preserve much of the performance on the Gleason patterns. On the explanations and the sub-explanations, this approach performed worse than the other methods.

We suspect this behavior to stem from our loss formulation, which, when taking the gradient, gives a positive reinforcement to each explanation that constitutes to the correct label of the higher hierarchy level, therefore also enforcing wrong labels on the lower levels of the hierarchy, consequently leading to higher entropy predictions.


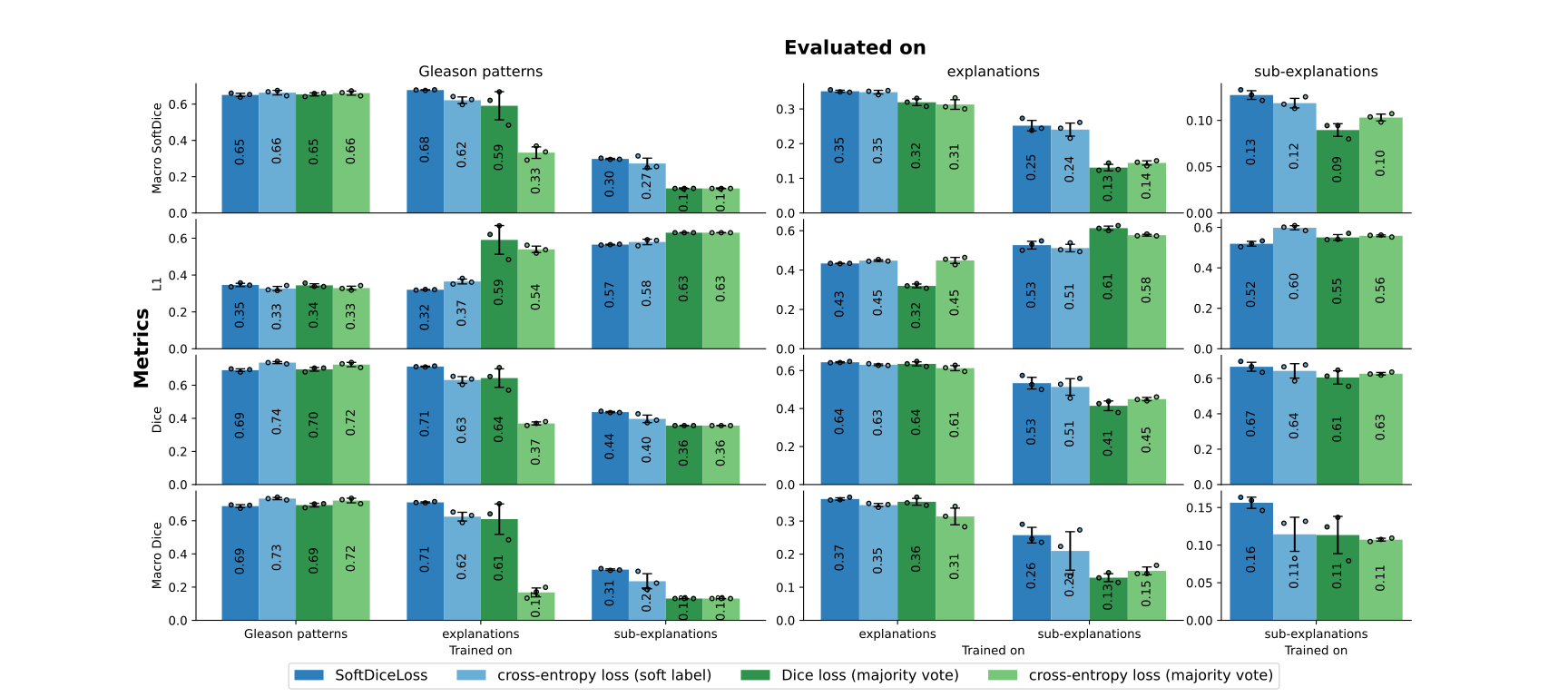


**Supplementary Fig. 2**: **Extended Results.** Barplot of the results for models trained with different loss functions evaluated and trained on the Gleason patterns, the explanations as well as the original sub-explanations. Using our ontology we map the labels upwards in the ontology, comparing the performance of models trained at lower levels of the ontology with models directly trained on the Gleason patterns and explanations. We plot the mean and the standard deviation of $n=3$models trained with the same hyperparameters but different seeds, and repeat the mean inside of the bar plots. Source data are provided as a Source Data file.

### Numerical Results

**Supplementary Table 11**: **Numerical extended results.** Results for models trained with different loss functions evaluated on Gleason patterns, and and trained on Gleason patterns, explanations and sub-explanations. Arrows indicate the direction of increasing performance. Each setting was trained 3 times, we report the mean and standard deviation for each metric. Abbreviations: ‘patterns’ : Gleason patterns, ‘expl.’: explanations.

| Task | | | Metrics | | | | | | | |
| --- | --- | --- | --- | --- | --- | --- | --- | --- | --- | --- |
|  |  |  | soft label | | | | majority vote | | | |
|  |  |  | Macro SoftDice ↑ | | $L_{1}$ ↓ | | Dice ↑ | | Macro Dice ↑ | |
| Evaluated | Trained | Loss | mean | std | mean | std | mean | std | mean | std |
| patterns | patterns | SoftDiceLoss | 0.651 | 0.011 | 0.346 | 0.011 | 0.691 | 0.010 | 0.688 | 0.011 |
| patterns | patterns | Dice loss (majority vote) | 0.653 | 0.010 | 0.344 | 0.011 | 0.696 | 0.014 | 0.694 | 0.014 |
| patterns | patterns | cross-entropy loss (majority vote) | 0.661 | 0.014 | 0.330 | 0.013 | 0.725 | 0.016 | 0.722 | 0.017 |
| patterns | patterns | cross-entropy loss (soft label) | 0.663 | 0.015 | 0.327 | 0.015 | 0.736 | 0.008 | 0.733 | 0.008 |
| patterns | expl. | SoftDiceLoss | 0.677 | 0.002 | 0.320 | 0.002 | 0.713 | 0.003 | 0.711 | 0.004 |
| patterns | expl. | Dice loss (majority vote) | 0.591 | 0.095 | 0.591 | 0.095 | 0.643 | 0.069 | 0.610 | 0.112 |
| patterns | expl. | TreeLoss(SoftDiceLoss) | 0.642 | 0.015 | 0.642 | 0.015 | 0.678 | 0.015 | 0.675 | 0.017 |
| patterns | expl. | cross-entropy loss (majority vote) | 0.333 | 0.039 | 0.540 | 0.021 | 0.369 | 0.012 | 0.168 | 0.033 |
| patterns | expl. | cross-entropy loss (soft label) | 0.621 | 0.023 | 0.365 | 0.016 | 0.632 | 0.025 | 0.625 | 0.032 |
| patterns | sub-expl. | SoftDiceLoss | 0.298 | 0.004 | 0.565 | 0.002 | 0.438 | 0.005 | 0.305 | 0.006 |
| patterns | sub-expl. | Dice loss (majority vote) | 0.135 | 0.000 | 0.630 | 0.000 | 0.356 | 0.000 | 0.131 | 0.000 |
| patterns | sub-expl. | TreeLoss(SoftDiceLoss) | 0.642 | 0.009 | 0.354 | 0.007 | 0.685 | 0.010 | 0.681 | 0.012 |
| patterns | sub-expl. | cross-entropy loss (majority vote) | 0.135 | 0.000 | 0.630 | 0.000 | 0.356 | 0.000 | 0.131 | 0.000 |
| patterns | sub-expl. | cross-entropy loss (soft label) | 0.273 | 0.036 | 0.580 | 0.018 | 0.397 | 0.028 | 0.235 | 0.056 |

**Supplementary Table 12**: **Numerical extended results.** Results for models trained with different loss functions evaluated and and trained on explanations and sub-explanations. Arrows indicate the direction of increasing performance. Each setting was trained 3 times, we report the mean and standard deviation for each metric. Abbreviations: ‘patterns’ : Gleason patterns, ‘expl.’: explanations

| Task | | | Metrics | | | | | | | |
| --- | --- | --- | --- | --- | --- | --- | --- | --- | --- | --- |
|  |  |  | soft label | | | | majority vote | | | |
|  |  |  | Macro SoftDice ↑ | | $L_{1}$ ↓ | | Dice ↑ | | Macro Dice ↑ | |
| Evaluated | Trained | Loss | mean | std | mean | std | mean | std | mean | std |
| expl. | expl. | SoftDiceLoss | 0.351 | 0.004 | 0.433 | 0.001 | 0.643 | 0.004 | 0.367 | 0.005 |
| expl. | expl. | Dice loss (majority vote) | 0.319 | 0.012 | 0.319 | 0.012 | 0.635 | 0.013 | 0.358 | 0.013 |
| expl. | expl. | TreeLoss(SoftDiceLoss) | 0.333 | 0.010 | 0.333 | 0.010 | 0.562 | 0.023 | 0.273 | 0.020 |
| expl. | expl. | cross-entropy loss (majority vote) | 0.313 | 0.017 | 0.449 | 0.019 | 0.613 | 0.016 | 0.314 | 0.031 |
| expl. | expl. | cross-entropy loss (soft label) | 0.348 | 0.007 | 0.448 | 0.005 | 0.630 | 0.005 | 0.348 | 0.006 |
| expl. | sub-expl. | SoftDiceLoss | 0.252 | 0.019 | 0.526 | 0.024 | 0.534 | 0.037 | 0.257 | 0.029 |
| expl. | sub-expl. | Dice Loss (majority vote) | 0.131 | 0.011 | 0.613 | 0.013 | 0.414 | 0.031 | 0.129 | 0.015 |
| expl. | sub-expl. | TreeLoss(SoftDiceLoss) | 0.313 | 0.007 | 0.467 | 0.006 | 0.538 | 0.013 | 0.246 | 0.023 |
| expl. | sub-expl. | cross-entropy loss (soft label) | 0.240 | 0.023 | 0.512 | 0.023 | 0.514 | 0.054 | 0.210 | 0.071 |
| expl. | sub-expl. | cross-entropy loss (majority vote) | 0.144 | 0.007 | 0.577 | 0.005 | 0.450 | 0.010 | 0.149 | 0.014 |
| sub-expl. | sub-expl. | SoftDiceLoss | 0.127 | 0.006 | 0.519 | 0.015 | 0.666 | 0.031 | 0.156 | 0.009 |
| sub-expl. | sub-expl. | TreeLoss(SoftDiceLoss) | 0.129 | 0.004 | 0.558 | 0.012 | 0.590 | 0.010 | 0.105 | 0.012 |
| sub-expl. | sub-expl. | cross-entropy loss (soft label) | 0.119 | 0.006 | 0.598 | 0.013 | 0.643 | 0.050 | 0.114 | 0.028 |
| sub-expl. | sub-expl. | Dice loss (majority vote) | 0.090 | 0.008 | 0.550 | 0.018 | 0.605 | 0.046 | 0.113 | 0.030 |
| sub-expl. | sub-expl. | cross-entropy loss (majority vote) | 0.103 | 0.005 | 0.558 | 0.005 | 0.627 | 0.009 | 0.107 | 0.002 |

### Comparison of alternative architectures

To validate the choice of the U-Net architecture, with an EfficientNet-B4 encoder, we trained a suite of alternative models and compared their performances.
We varied the encoders, e.g. replacing the EfficientNet-B4 with an DenseNet-201[^1^](https://paperpile.com/c/KpWYi9/nvbv) encoder (this concrete DenseNet was chosen as it has a comparable number of parameters). Furthermore, we varied the segmentation architecture, using a UNet++[^2^](https://paperpile.com/c/KpWYi9/ZpiUw) and a DeepLabV3+[^3^](https://paperpile.com/c/KpWYi9/a8t7), both with an identical EfficientNet-B4 encoder. Finally, we tested the older FCN[^4^](https://paperpile.com/c/KpWYi9/0pawm) for semantic segmentation with an ResNet-50 encoder.

We trained all models to segment the explanations, using the DICE loss for the majority voted hard-labels or the SoftDICELoss for the soft-label approach. The results are reported in Supplementary Table 13.

**Supplementary Table 13**: **Results with additional architectures.** Models trained with the SoftDICE loss used soft-labels, while DICE-trained models used hard-labels (leaving out pixels without majority vote). All models were trained on the explanations and evaluated on the explanations and the Gleason level. Our original results from the paper are shaded in light blue. The best result per column is marked in **bold.**

| Loss function | Architecture | Encoder | Gleason | | | | Explanations | | | |
| --- | --- | --- | --- | --- | --- | --- | --- | --- | --- | --- |
|  |  |  | Macro DICE | | Macro SoftDICE | | Macro DICE | | Macro SoftDICE | |
|  |  |  | mean | std | mean | std | mean | std | mean | std |
| SoftDICE | UNet | EfficientNetB4 | **0.711** | **0.004** | **0.677** | **0.001** | **0.367** | **0.005** | **0.351** | **0.004** |
| SoftDICE | UNet | DenseNet-201 | 0.691 | 0.011 | 0.657 | 0.008 | 0.356 | 0.003 | 0.333 | 0.008 |
| SoftDICE | UNet++ | EfficientNetB4 | 0.662 | 0.035 | 0.629 | 0.029 | 0.343 | 0.009 | 0.319 | 0.012 |
| SoftDICE | FCN | ResNet50 | 0.680 | 0.007 | 0.651 | 0.008 | 0.366 | 0.010 | 0.343 | 0.007 |
| SoftDICE | DeepLabV3+ | EfficientNetB4 | 0.672 | 0.013 | 0.647 | 0.011 | 0.360 | 0.011 | 0.330 | 0.006 |
| DICE | UNet | EfficientNetB4 | 0.610 | 0.112 | 0.590 | 0.095 | 0.358 | 0.013 | 0.319 | 0.012 |
| DICE | UNet | DenseNet-201 | 0.521 | 0.082 | 0.524 | 0.071 | 0.356 | 0.004 | 0.313 | 0.004 |
| DICE | UNet++ | EfficientNetB4 | 0.533 | 0.076 | 0.525 | 0.060 | 0.354 | 0.012 | 0.315 | 0.010 |
| DICE | FCN | ResNet50 | 0.517 | 0.033 | 0.527 | 0.028 | 0.346 | 0.003 | 0.311 | 0.001 |
| DICE | DeepLabV3+ | EfficientNetB4 | 0.581 | 0.002 | 0.579 | 0.014 | 0.342 | 0.009 | 0.309 | 0.001 |

As demonstrated by our results, the use of different architectural variants yields largely comparable performance. We initially selected the EfficientNet architecture due to its strong performance and high computational efficiency, outperforming alternatives such as DenseNet and ResNet in this regard [^5^](https://paperpile.com/c/KpWYi9/cqlpK). As expected, the EfficientNet-based model outperformed the version using a DenseNet-201 encoder. Similarly, prior studies have shown through comprehensive benchmarking that modifications to the U-Net architecture, such as UNet++, UNet3+, and others, often achieve similar performance levels [^6^](https://paperpile.com/c/KpWYi9/ZOiST)^,^[^6,7^](https://paperpile.com/c/KpWYi9/ZOiST+8Uxtz). This observation is consistent with our findings, where both DeepLabV3+ and UNet++ failed to improve upon our fine-tuned U-Net baseline. The original Fully Convolutional Network (FCN), which lacks a dedicated decoder path and predates the U-Net architecture, exhibited inferior segmentation performance, as expected.

All experiments were conducted using the same hyperparameters, due to constraints in time and computational resources. However, based on our results and the literature, we do not anticipate that alternative encoder or decoder architectures would yield substantially better performance under the same conditions.

###

### Additional Study: Application on Whole Slide Images

For the external validation, we created a pipeline applying the GleasonXAI to whole slide images using a sliding window approach. We test our model against pixel wise Gleason pattern (GP) annotations by predicting explanations and remapping them to their respective Gleason pattern.

First, we tested GleasonXAI on the AGGC challenge [^8^](https://paperpile.com/c/KpWYi9/YpBGg). The dataset consists of three subsets, with 45, 16, and 67 test images, and 105, 37, and 144 training images, respectively. The first and third subsets contain whole mount images, whereas the second consists of biopsy images. All subsets are scanned with an Akoya scanner. The whole mounts of the third subset are also scanned by up to five additional, different scanners [^8^](https://paperpile.com/c/KpWYi9/YpBGg). The challenge contains annotations for GP3, GP4, GP5, normal and stroma tissue. The annotations are not exhaustive, so in the assessment for the challenge, the weighted-average F1-score is calculated on the annotated area only. Since our model doesn’t predict normal and stroma, the F1 scores are calculated in the whole annotated area for GP3, GP4, and GP5, and for a class representing the union of the remaining classes. Additionally, we applied the GleasonXAI to the whole images to demonstrate the general performance.

For a second dataset, we evaluated GleasonXAI on DiagSet Part A [^9^](https://paperpile.com/c/KpWYi9/VRlXo), which comprises 425 biopsy WSIs annotated by three pathologists for scan background, tissue background, healthy tissue, artifacts, and Gleason patterns 1 to 5 within outlined regions. To match the model’s outputs, we collapsed these labels into GP3, GP4, GP5, and an “Other” category. Because the original DiagSet evaluation focused on binary cancer-versus-noncancer decisions and patch-level classification, a direct comparison was not possible. Instead, we computed per-class F1 scores over all annotated pixels and applied GleasonXAI to the whole image for qualitative visualization.

To use the images for GleasonXAI, they were reformatted to a common physical pixel side length of 1.392 $\frac{\mathsf{\mu m}}{\mathsf{px}}$. A sliding window approach was applied using a 50% overlap and patch sizes of 512x512 px². The patches were recombined using an average merger. Patches without tissue were skipped during inference. For the identification of the region of interest, we tested the inclusion of a patch-wise tissue subtyper trained on colon tissue for the detection of tumorous tissue [^10^](https://paperpile.com/c/KpWYi9/ZUUnV), since GleasonXAI was trained mainly on such tumor tissue, but no benefit was found. The predictions were mapped to Gleason grades, and the final prediction generated by taking the class with the highest probability per pixel. The predictions were evaluated on the tissue area determined through Otsu thresholding as described in our paper. The metrics were determined on the annotated area only.

To test transferability to other domains, and due to the difference in labels, we chose to apply GleasonXAI without further transfer training.

As shown in Supplementary Table 14 and Supplementary Table 15, the GleasonXAI achieved constantly high F1 scores in GP 3, ranging from 0.667 to 0.722, and notable performance for GP 4 with F1 scores up to 0.704 on the AGGC’s whole mount images (AGGC part 1 and 3). It further showed very good performance in the differentiation between tumorous and non-tumorous tissue, as demonstrated by the good F1 scores of the other tissues in the evaluation area. This can also be seen in the visualizations of the results in Supplementary Fig. 3, where few areas outside the non-exhaustive ground truth labels are classified with any Gleason pattern, even without further adjustments and pre-selection of tumor tissue. These results highlight the model's robustness in identifying intermediate-grade tumors. In addition, the precision values for GP3 and GP4 were remarkably high (up to 0.960 for GP4 in part 3), suggesting that GleasonXAI is particularly conservative in assigning these labels.

A subanalysis of AGGC part 3 revealed a low difference in performance between the scanners, with the standard deviation in the F1 scores ranging from 0.006 to 0.055 between the results on the scanner subsets, depending on the class, which indicates robustness against changes in scanners.


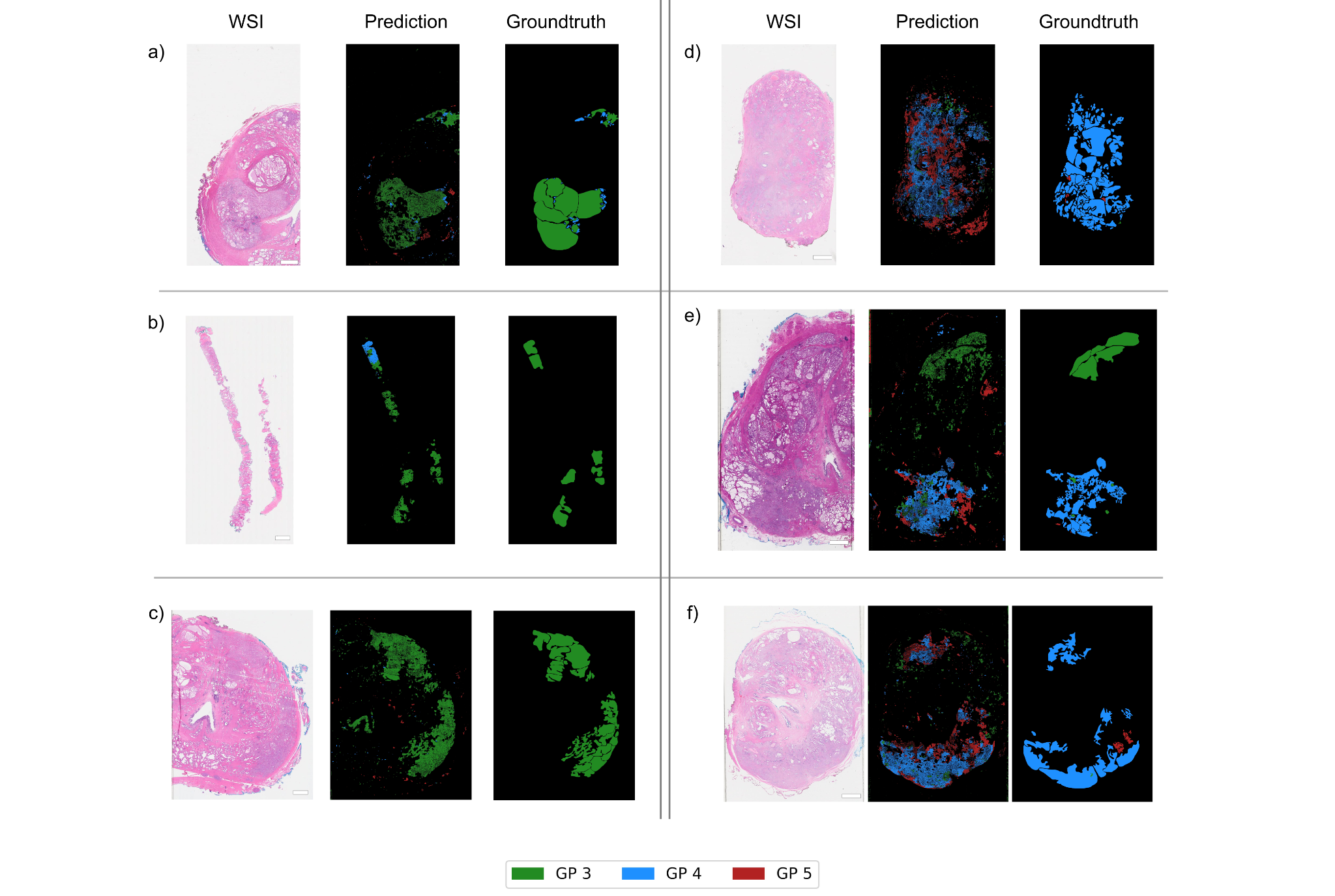


**Supplementary Fig. 3: Predictions on AGGC test set.** Examples of the segmentation results of the GleasonXAI on the AGGC data, showing the WSI (micron adjusted), the background-adjusted prediction created by the GleasonXAI within and outside of the evaluation area, and the ground truth label within the evaluation area. Panels a) to c) show examples of good agreement between the prediction and the ground truth. Overall, as presented in panels d) to f), there is a tendency for over segmentation of Gleason pattern 5. Scale bar corresponds to 600µm.

We observe pronounced oversegmentation of GP 5. Because this pattern is rare in the training set for the GleasonXAI, we used a macro soft‑Dice loss, which averages the Dice error over all classes and therefore gives the minority class the same weight as the majority ones. This equal weighting boosts the gradient signal for GP 5 and was necessary for the model to learn the pattern in our dataset, but it also increases the risk of false‑positive GP 5 predictions when the decision threshold or calibration are not perfectly tuned. Under the additional domain shift between the training data and the new cohort, this sensitivity manifests as oversegmentation of GP 5, reflected by high recall but low precision, resulting in a low per‑class F1 score. A general decrease in performance was expected, as domain-shifts are a well-known phenomenon in machine learning. To increase the transferability, future work could use foundation models pre-trained on a large variety of images from varying sources and collect more diverse data from more scanners and clinics.

Nonetheless, most of the misclassification is into adjacent classes or tissue not annotated with Gleason patterns 3, 4, or 5. This is similar to the results found in our dataset, and can be further inspected in Supplementary Fig. 4, where the row-normalized confusion matrices for pixels of all images of the AGGC dataset is presented. As can be seen, there is almost no misdiagnosis between GP 3 and GP 5.


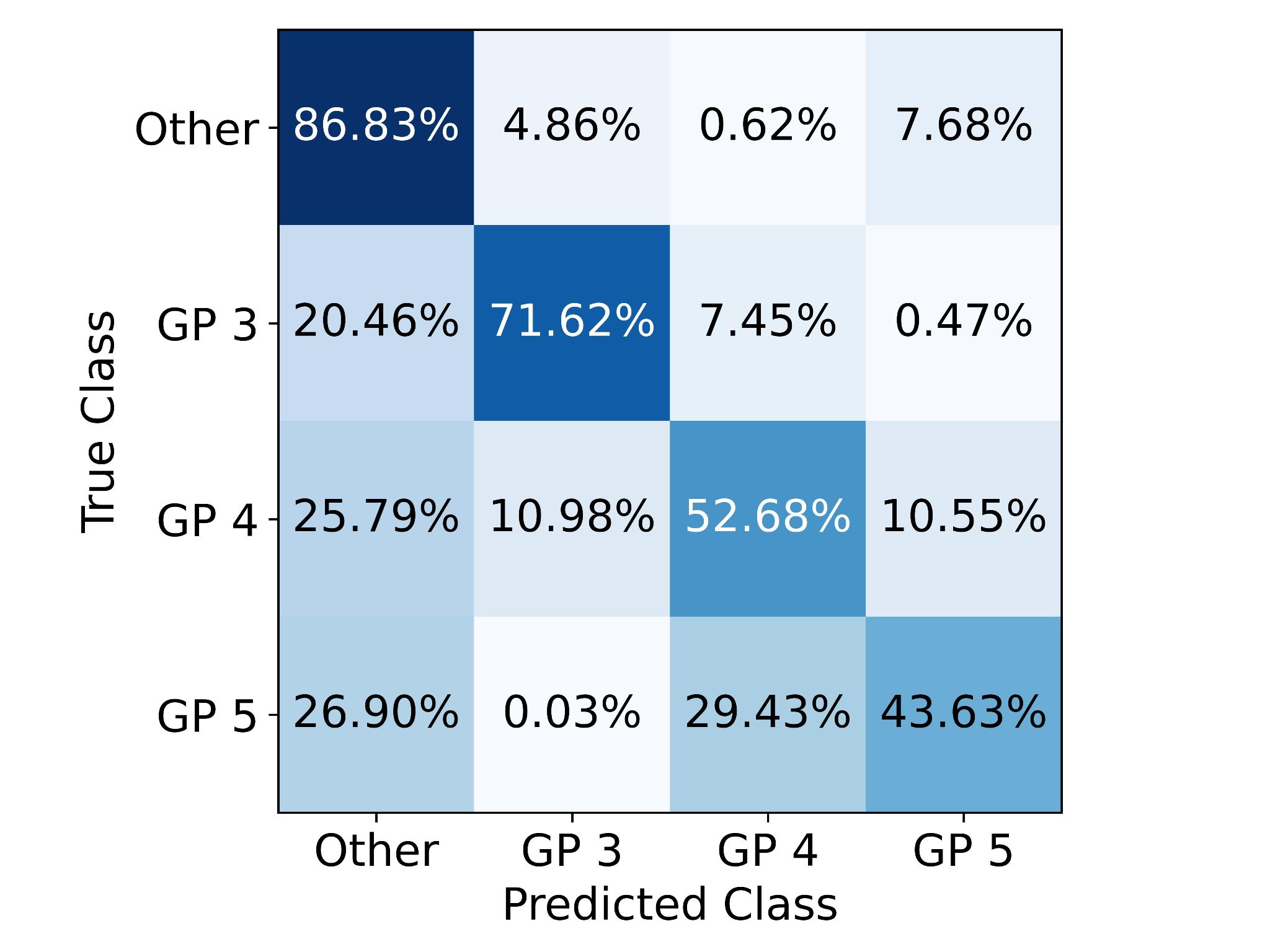


**Supplementary Fig. 4: Confusion matrix for AGGC predictions.** Confusion matrices for pixels in the evaluation areas of all images in the AGGC dataset.

Overall, GleasonXAI performs better on the whole mount images (AGGC part 1 and 3) than on biopsy images in AGGC part 2 and DiagSet (see Supplementary Table 14 and 15), which can likely be attributed to the fragmented nature of biopsies. Biopsies include more border areas, which often induce more artefacts. They contain a higher variety of tissue types, and are less likely to contain larger sheets of similar tissue compared to whole mount images, thus, they are less similar to TMA cores and therefore more likely to contain out-of-distribution patches. For the use of GleasonXAI on biopsies we therefore recommend training the model on a transfer dataset before use.

In conclusion, even in the absence of domain adaptation, GleasonXAI has demonstrated a strong baseline performance in GP3 and GP4, especially on whole mount images. The AI is usable without previous tumor tissue detection in the preprocessing, but might benefit from it for the Gleason 5 patterns, if a segmentation rather than a patch classification approach is used. Future improvements on the AI should focus on enhancing performance for rare patterns like GP5, and on the extension of the training data with common artefacts, which will further strengthen the model’s diagnostic utility in a non-TMA use case.

**Supplementary Table 14: Results on AGGC sets.** F1-score, Precision, and Recall achieved by the GleasonXAI on the AGGC test set (Subset 1), and AGGC training set (Subset 2) for each of the Gleason patterns (GP) and the combined class of other annotated tissue types on the tissue pixels in the annotated areas of the images.

| Subset 1 | | | | | | | | | | | | |
| --- | --- | --- | --- | --- | --- | --- | --- | --- | --- | --- | --- | --- |
| Metric | AGGC part 1 | | | | AGGC part 2 | | | | AGGC part 3 | | | |
|  | Other | GP3 | GP4 | GP5 | Other | GP3 | GP4 | GP5 | Other | GP3 | GP4 | GP5 |
| F1  score | 0.791 | 0.722 | 0.622 | 0.354 | 0.878 | 0.667 | 0.191 | 0.057 | 0.708 | 0.720 | 0.704 | 0.025 |
| Preci-sion | 0.698 | 0.679 | 0.851 | 0.284 | 0.814 | 0.847 | 0.295 | 0.030 | 0.593 | 0.756 | 0.960 | 0.013 |
| Recall | 0.913 | 0.771 | 0.490 | 0.470 | 0.953 | 0.552 | 0.141 | 0.614 | 0.876 | 0.687 | 0.556 | 0.391 |
| Subset 2 | | | | | | | | | | | | |
| Metric | AGGC part 1 | | | | AGGC part 2 | | | | AGGC part 3 | | | |
|  | Other | GP3 | GP4 | GP5 | Other | GP3 | GP4 | GP5 | Other | GP3 | GP4 | GP5 |
| F1  score | 0.705 | 0.673 | 0.580 | 0.304 | 0.899 | 0.706 | 0.441 | 0.079 | 0.677 | 0.779 | 0.711 | 0.058 |
| Preci-  sion | 0.579 | 0.690 | 0.850 | 0.250 | 0.857 | 0.795 | 0.570 | 0.042 | 0.582 | 0.803 | 0.910 | 0.030 |
| Recall | 0.896 | 0.660 | 0.440 | 0.389 | 0.945 | 0.635 | 0.359 | 0.636 | 0.807 | 0.756 | 0.585 | 0.701 |

**Supplementary Table 15: Results on DiagSet.** F1-score, Precision, and Recall achieved by the GleasonXAI on the AGGC test set (Subset 1), AGGC training set (Subset 2) and on the DiagSet for each of the Gleason patterns (GP) and the combined class of other annotated tissue types on the tissue pixels in the annotated areas of the images.

| Metric | Other | GP3 | GP4 | GP5 |
| --- | --- | --- | --- | --- |
| F1  score | 0.911 | 0.379 | 0.446 | 0.310 |
| Preci-  sion | 0.916 | 0.347 | 0.600 | 0.211 |
| Recall | 0.907 | 0.417 | 0.256 | 0.575 |

## Comparison of Pathologist-recognized Morphological Patterns and Predicted Patterns

As with other machine learning methods our method is not immune to shortcut learning [^11^](https://paperpile.com/c/KpWYi9/YtbEW), the integration of causally irrelevant features into the learned decision.

However, the approach adopted in this study has the advantage over direct prediction of Gleason patterns in that the predicted features are interpretable by experts through the use of the ISUP guideline-based ontology. Therefore, the correctness of our predictions is directly verifiable by pathologists, leading to an increased inherent transparency, avoiding pitfalls of previously used post-hoc explainability methods.

For the GleasonXAI, the areas predicted by the model aligned well with the annotated areas of the experts, showing similarities in their structures. As demonstrated in Supplementary Fig. 5, classes associated with glands are predicted in areas with glands, whereas the classes defined by the structure of cell formations show no or fewer glands. This finding suggests that the feature detection is at least partially guided by structures in the tissue.


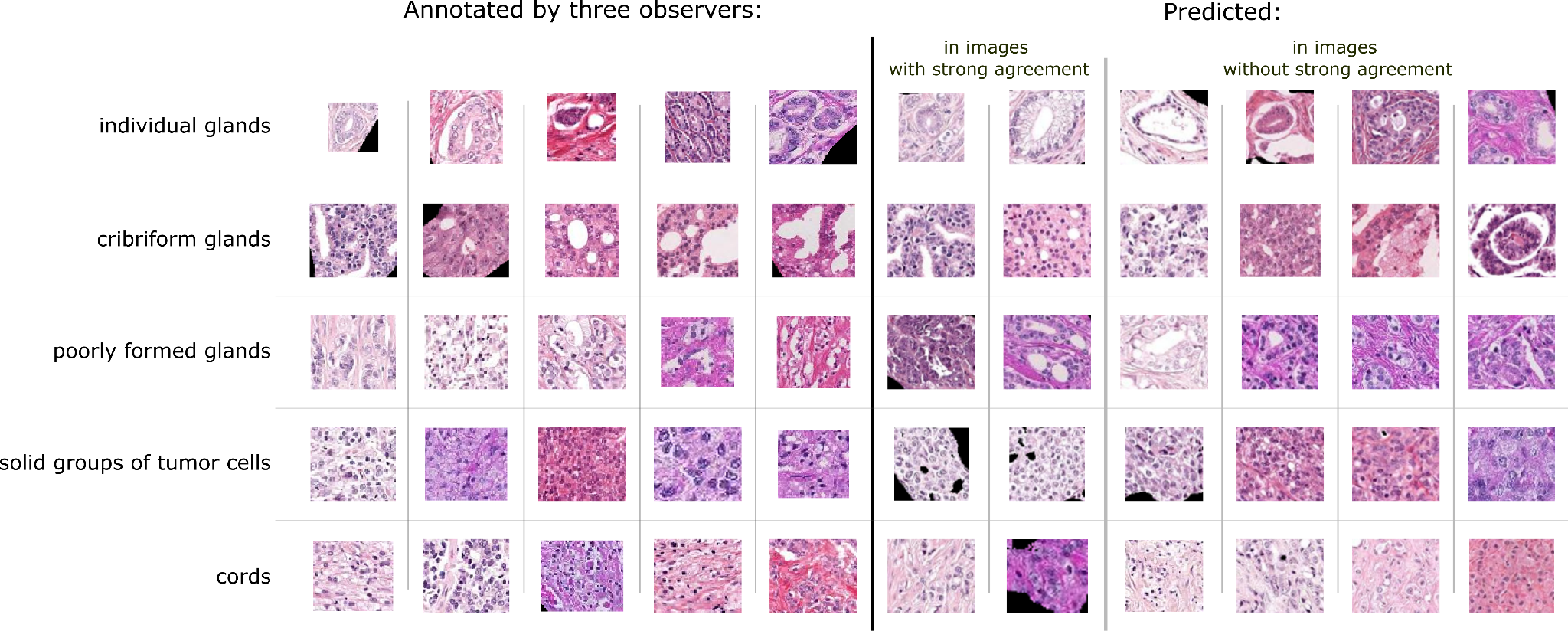


**Supplementary Fig. 5: Tissue Examples.** Patches of TMAs that were assigned an explanation by three annotators (left), and patches assigned the explanation by the GleasonXAI (right), either in images where there was also a three rater agreement of the explanation within image or in images without three rater agreement on the explanation.

## Data Selection


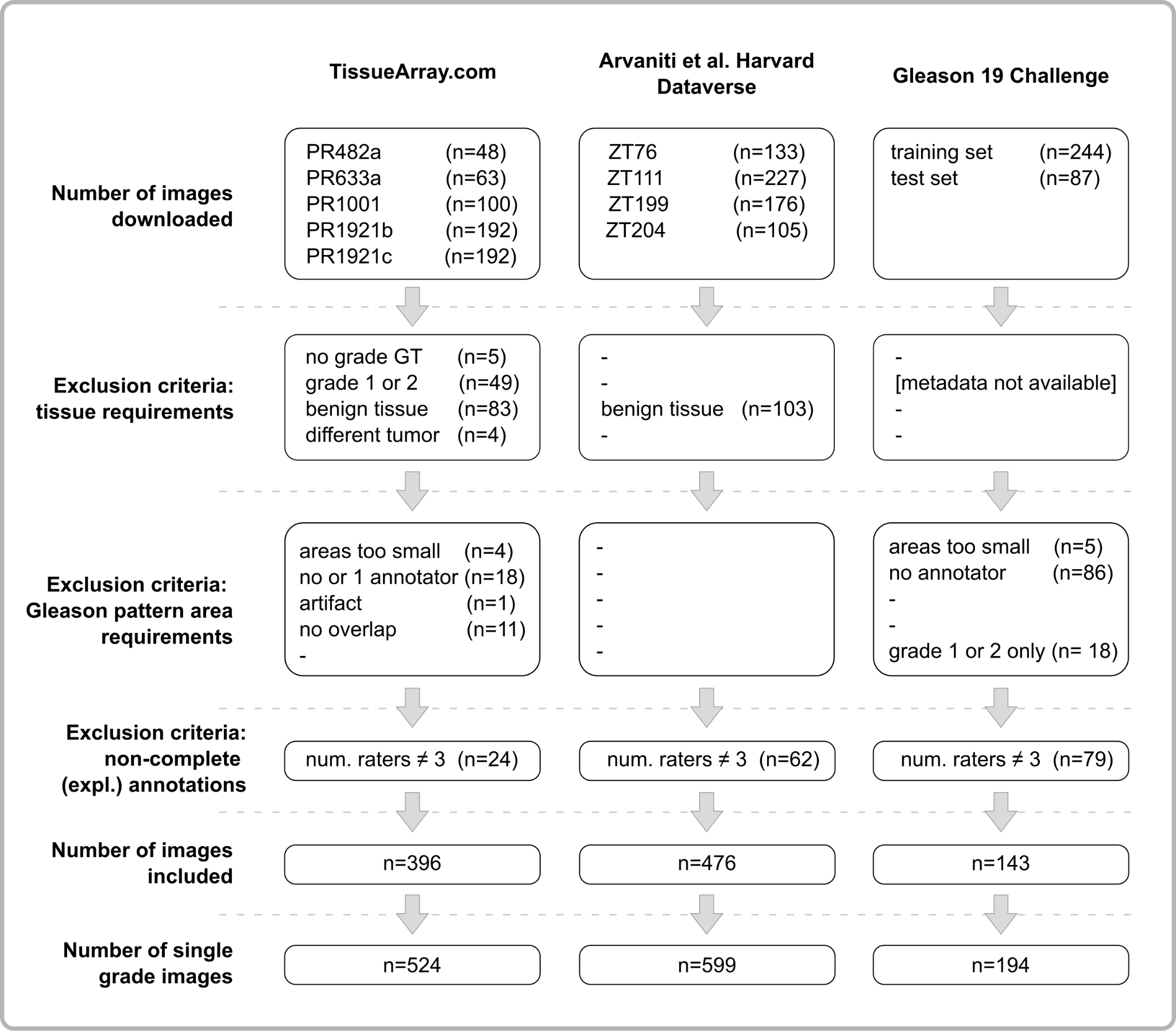


**Supplementary Fig. 6:** Slide selection process for the three datasets. ‘-’: no images excluded by this filter, ‘GT’: ground truth.

As shown in the Supplementary Fig. 6, the majority of images excluded before the explanatory annotations were removed since no annotations were provided by the Gleason 19 challenge. They are part of the test set, for which the annotations are not publicly available and we did not consider them for labeling by our pathologists, since the amount of data available for explanation annotations was deemed sufficient. It was determined that further analysis of these images was not required, as we anticipated that their characteristics would closely resemble those of the training set from the Gleason 19 challenge. Further 18 of the images were excluded because they only contained Gleason patterns 1 and 2, which were not relevant for our approach and are not used in practice anymore, therefore eliminating the need for further analysis.

Of the remaining 39 excluded images, most were filtered due to missing annotators or lack of overlap in the Gleason grade annotations. The images with missing annotators were analyzed, but there were no consistencies between the images. We therefore assume them to be skipped for time reasons.

Cases lacking overlap were discarded due to the inability of the pathologists to determine a reliable ground truth for these images. This would have resulted in the introduction of further noise into the already challenging training process. Moreover, our approach of fusing the Gleason grade annotations via STAPLE produces empty annotations on those cases, which could not be utilised. This would have necessitated the arbitrary selection of one of the annotations by the pathologist.

After the explanation annotation phase, further 165 images were removed due to lack of annotations by three annotators. To understand the potential biases introduced by the removal of images that were not fully annotated, we compared the distribution of the Gleason grade combinations provided in the image metadata for the excluded images with that for the explanation annotated dataset before removal (e.g. Grade 3+4). As shown in Supplementary Fig. 7, the majority of dropped images were those assigned a single Gleason grade (e.g. Gleason 3+3, 4+4, or 5+5), which aligns with the most frequently occurring grade combination in the annotated dataset. Overall, the distribution of removed images closely reflects that of the full dataset, as illustrated in Figure S.7c and Supplementary Table 16, where similar proportions of images were excluded across the various grade combinations. A notable exception is the Gleason Grade 3+5 combination, from which no images were removed. However, ‚due to its infrequent occurrence, this deviation is likely attributable to statistical variability rather than systematic bias.

**Supplementary Table 16:** **Ratio Comparison.** Number of TMAs with the given Gleason pattern in the dropped and in the overall annotated data, and the ratio of dropped to overall annotated TMAs.

| Gleason Pattern | TMAs with Gleason pattern in dropped data | TMAs with Gleason pattern in explanation annotated dataset | Ratio (in %) |
| --- | --- | --- | --- |
| 3 | 83 | 649 | 12.79 |
| 4 | 98 | 854 | 11.48 |
| 5 | 48 | 376 | 12.77 |

| a)  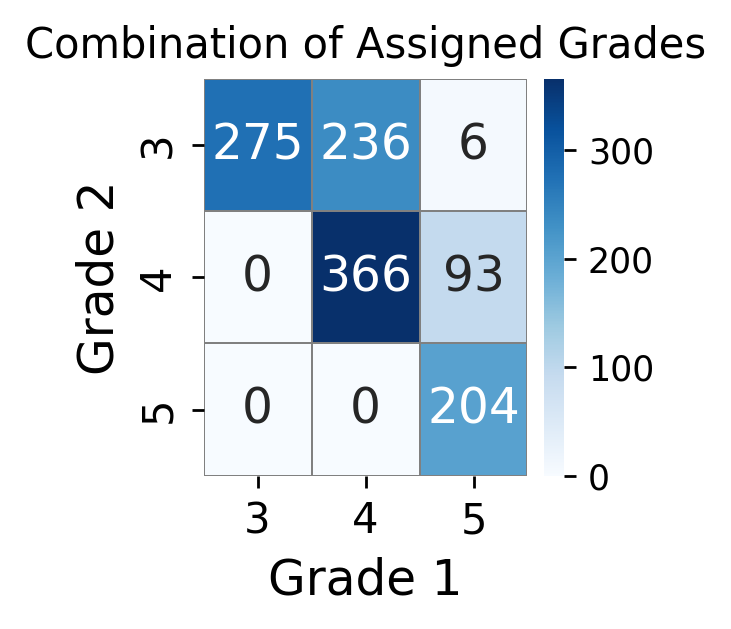 | b)  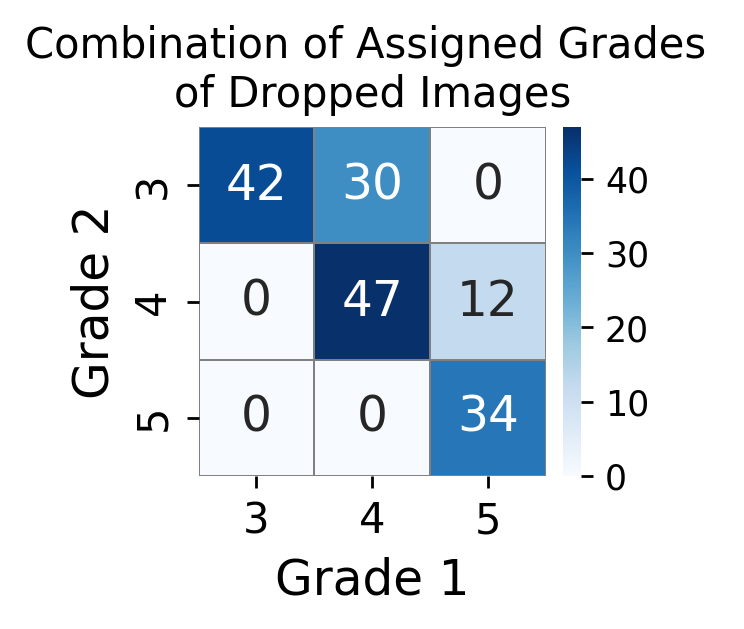 |
| --- | --- |
| c)  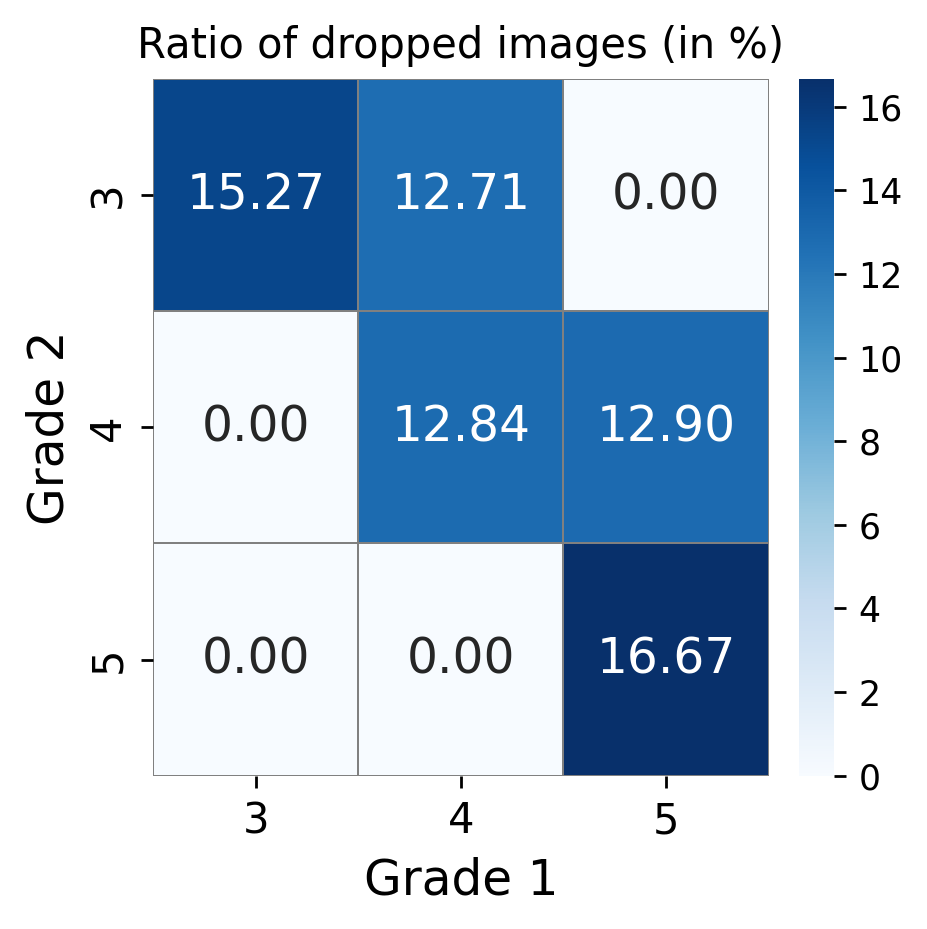 | |

**Supplementary Fig. 7: Assigned Grade Distribution in dropped images.** Distribution of assigned grades a) in the images before dropping images with more than three annotators, b) of the images dropped due to the number of raters and c) the percentage of dropped images per grade combination. Source data are provided as a Source Data file.

To evaluate whether the distribution of images across the Gleason Grade combinations changed significantly following the removal of the images, a $\chi^{2}$test [^12^](https://paperpile.com/c/KpWYi9/lkzG) was conducted at a significance level of $\alpha=0.05$. The test yielded a p-value of p = 0.377, indicating no statistically significant difference between the distributions. Consequently, the null hypothesis was retained, suggesting that the image removal process did not introduce a bias in the distribution of Gleason grades.

Since images were excluded due to missing annotations, we investigated the explanations provided by raters who did not skip these images. Specifically, we examined whether particular labels were disproportionately represented among the removed images. Our analysis revealed that no explanation was markedly overrepresented in the removals, and the overall distribution of explanation categories in the dropped subset closely mirrored that of the full dataset (see Supplementary Fig. 8 and Supplementary Table 17). An exception was observed for *Glomeruloid glands*, which compared to their overall occurrence appeared at a slightly higher relative frequency in the removed image. Nonetheless, this minimal increase does not represent a systematic problem.

| a)  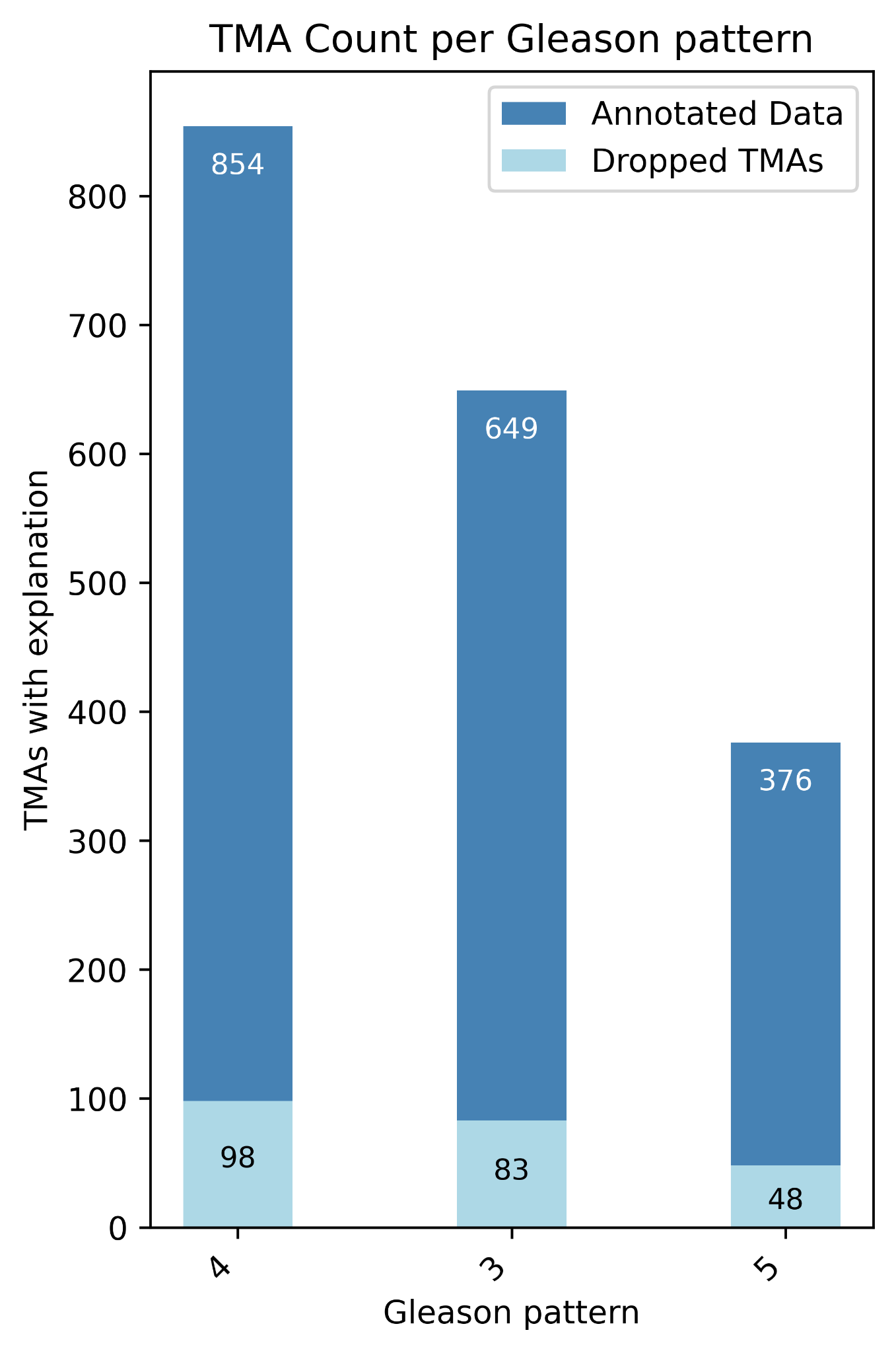 | b)  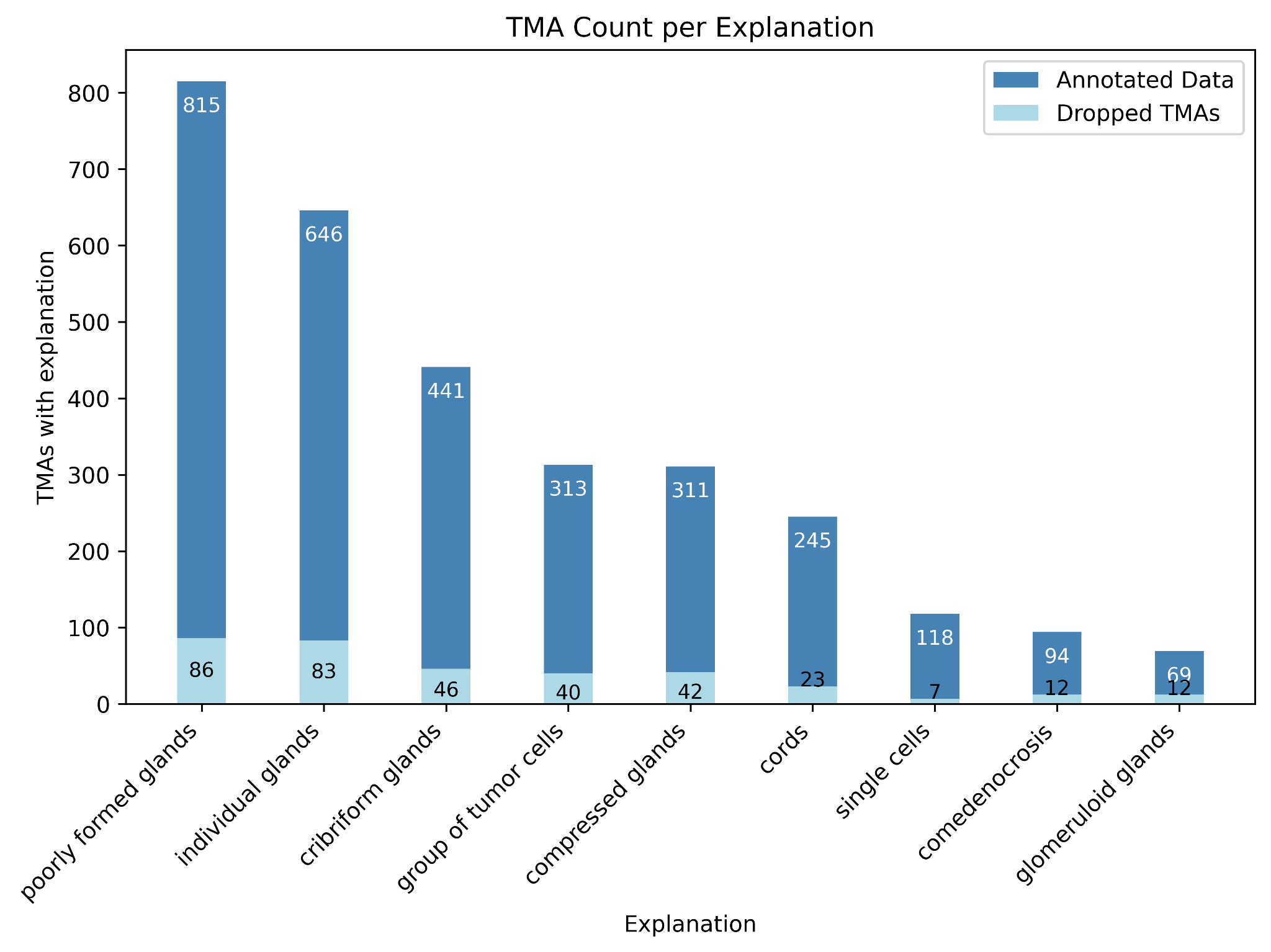 |
| --- | --- |

**Supplementary Fig. 8: Gleason pattern and Explanation occurrences.** Number of TMAs with occurrences of a) Gleason patterns and b) explanations in all the explanation annotated data and in the images dropped due to rater issues. Source data are provided as a Source Data file.

**Supplementary Table 17:** Number of TMAs with the given explanation in the dropped and in the overall annotated data, and the ratio of dropped to overall annotated TMAs.

| Explanation | TMAs with explanation in dropped data | TMAs with explanation in explanation annotated dataset | Ratio (in %) |
| --- | --- | --- | --- |
| Individual glands | 83 | 646 | 12.85 |
| Compressed glands | 42 | 311 | 13.50 |
| Poorly formed glands | 86 | 815 | 10.55 |
| Cribriform glands | 46 | 441 | 10.43 |
| Glomeruloid glands | 12 | 69 | 17.39 |
| Solid group of tumor cells | 40 | 313 | 12.77 |
| Single cells | 7 | 118 | 5.93 |
| cords | 23 | 245 | 9.39 |
| comedonecrosis | 12 | 94 | 12.77 |

#

# Supplementary Figures

## Gleason Pattern Masks


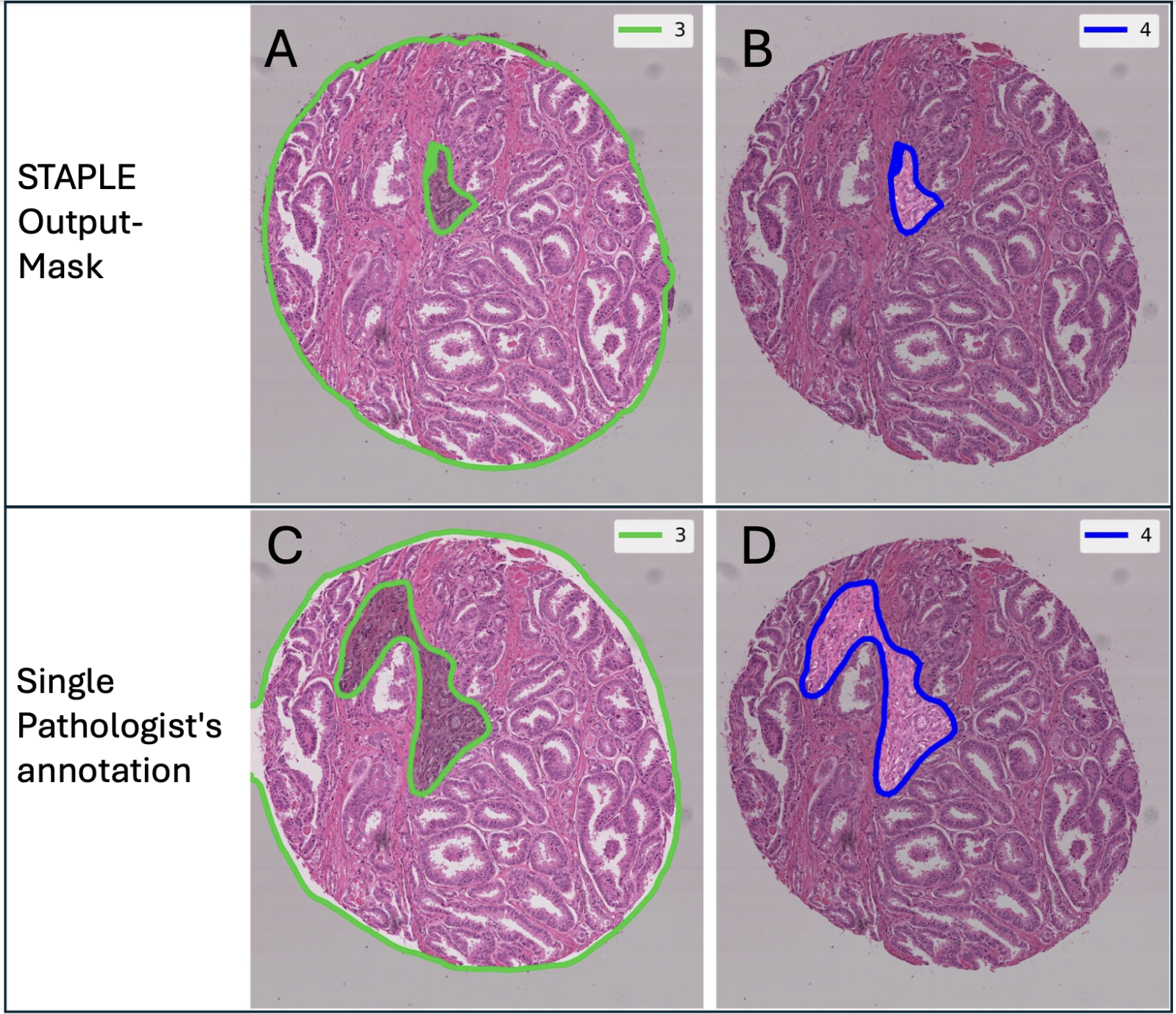


**Supplementary Fig. 9: Review of STAPLE output masks**. In cases where the output masks did not cover biological meaningful patterns, the pathologists' annotation, which was closest to the output masks, was taken. Here a representative example is presented. Gleason pattern grade 3 areas are outlined in green and Gleason pattern grade 4 areas are outlined in blue. Corresponding areas are highlighted in a brighter color. STAPLE output mask with an inaccurate Gleason pattern grade 3 (A) and 4 (B) area. Single pathologist annotation with adjusted area for Gleason grade 3 (C) and 4 (D).


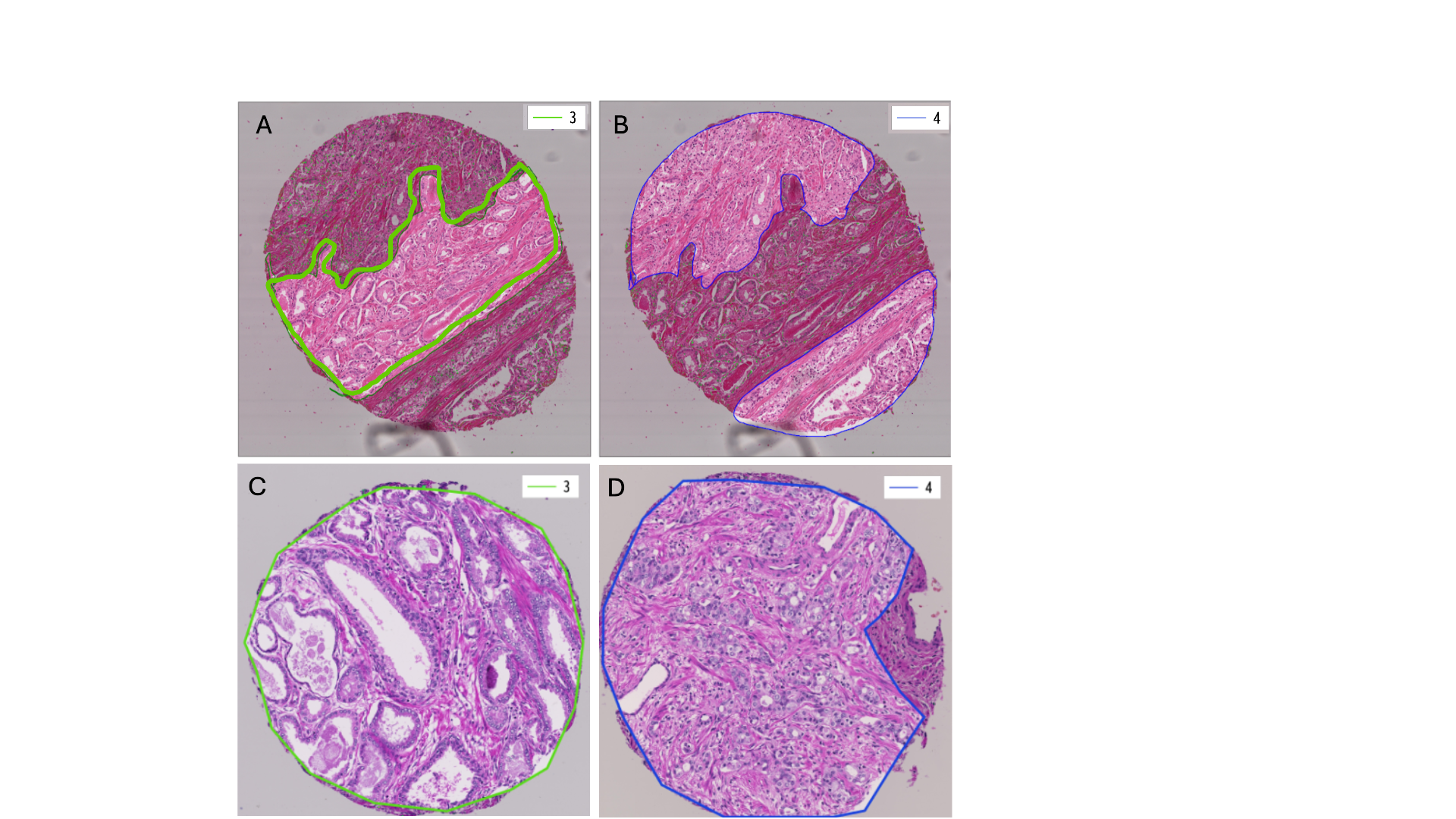


**Supplementary Fig. 10: Representation of overall Gleason pattern grade annotation:** TMA core images with highlighted Gleason grade areas for a case of Gleason grade 4+3. Areas of A) Gleason grade 4 were highlighted and outlined in blue and for B) Gleason grade 3 were outlined in a separate image in green to avoid confusion. Were the Gleason grades the same, only a single area was highlighted i.e. in C) for Gleason 3+3 in green and in D) for 4+4 in blue.

## Label Generation and Training Procedure


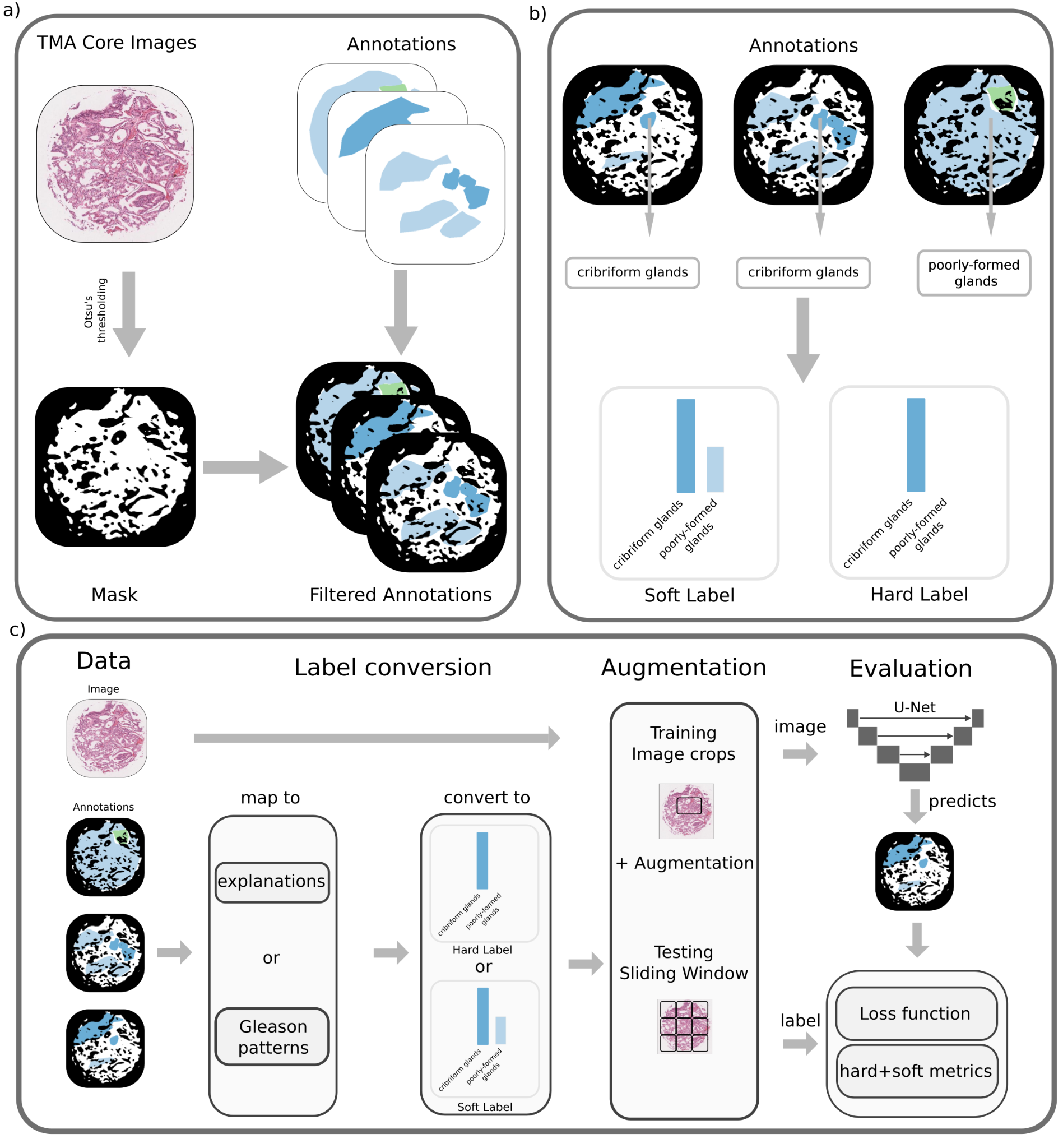


**Supplementary Figure 11: Training Procedure.** a) A foreground mask was extracted from the TMA core image by the use of Otsu’s thresholding, followed by morphological operations. The annotations were filtered with the foreground mask to distinguish background pixels from benign tissue pixels. b) The per-pixel annotations of the annotators were combined into a (soft) per-pixel label-distribution. Here the distribution over the explanations for one pixel (indicated by the arrows) is shown. c) Setup of our training pipeline. Models are either trained on the explanations or Gleason patterns, which is achieved through the remapping of explanation annotations. If we are using a soft label model, the three annotations are averaged pixel wise, otherwise a majority vote is taken, discarding undecided pixels for the hard metrics and hard loss functions. During training, images are augmented and random crops are taken, while during testing, only normalization is applied to the images together with a sliding window approach. The images are then fed through the U-Net to obtain a prediction, which is combined with the label mask to compute the loss, as well as the hard and soft metrics, either on Gleason pattern or explanation level.

## Data Split


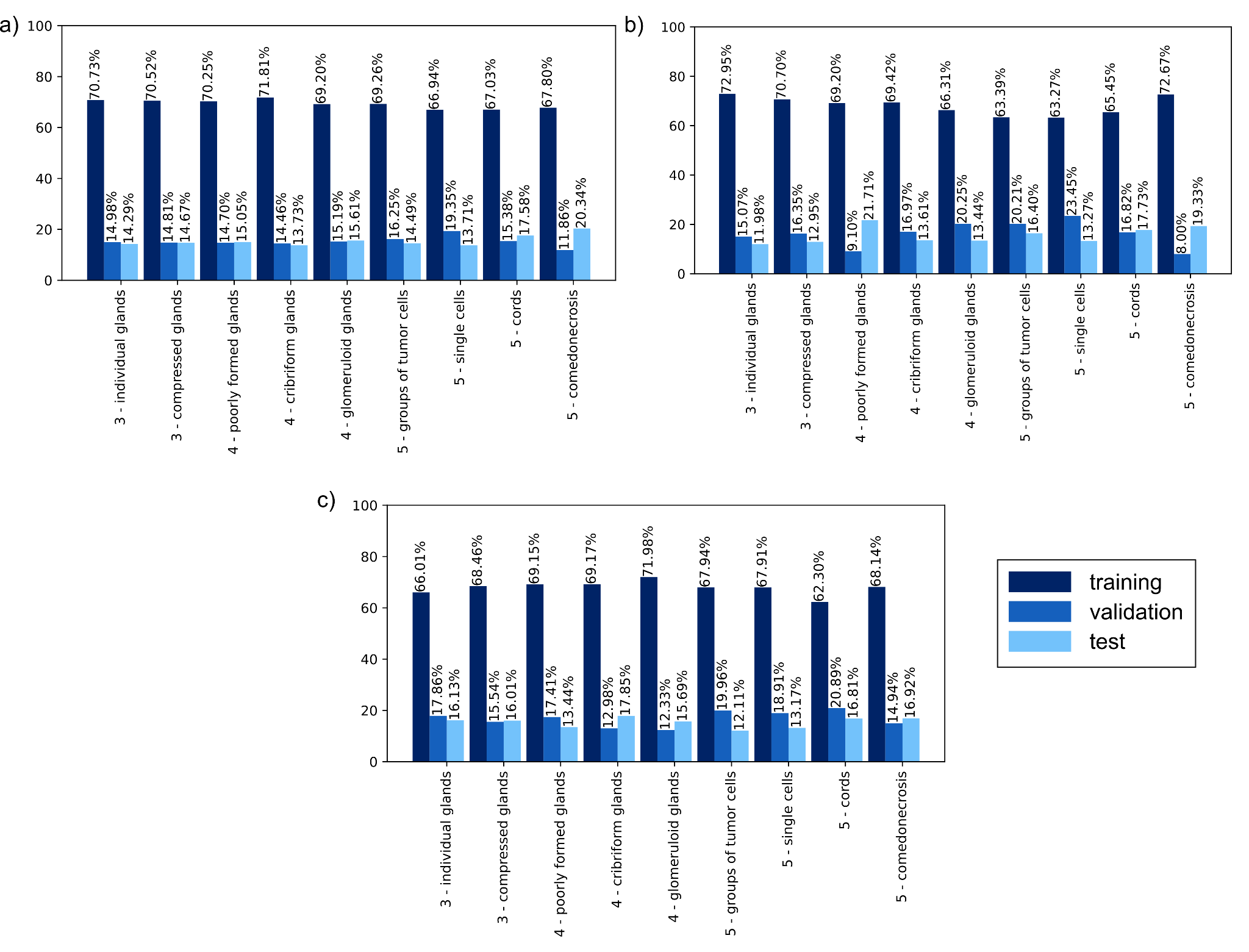


**Supplementary Fig. 12: Data Split.** Distribution of a) files, b) polygons, and c) pixels with explanations on the training, validation and test set. Source data are provided as a Source Data file.

# Supplementary Discussion

## Calibration Metric Discussion

When trained on the explanations, both the SoftDiceLoss and the soft label cross-entropy loss models achieved better or equal calibration on the Gleason patterns (see [Figure 5](https://docs.google.com/document/d/12SpMrcdW3LseAYLAlLt8UxaH2f4-exnavcTm3WOZLWQ/edit?pli=1#fig_metricresults)) compared to the majority-voted cross-entropy models. The Dice loss, on the other hand, provided the best calibration when trained and evaluated on the explanations.

Evaluated on the Gleason patterns, soft label approaches trained on the explanations consistently outperformed the majority-voted approaches in terms of calibration, and were equally well calibrated when trained on the Gleason Patterns directly.

We therefore observe an exception to the superior performance of the soft labels in the $L_{1}$-norm, where models trained on Dice loss surpass those using soft label approaches at explanation level. Since the $L_{1}$-norm is not calculated on a per-class basis, we hypothesize that the high-confidence predictions from models trained on hard labels better accommodate the large class imbalances, leading to improved performance. Consensus among pathologists was highest for the most common classes (see [Figure 4](https://docs.google.com/document/d/12SpMrcdW3LseAYLAlLt8UxaH2f4-exnavcTm3WOZLWQ/edit?pli=1#fig_pixelagreement)), resulting in labels that were largely equivalent to hard labels for these pixels. Conversely, the more conservative predictions from soft label approaches were less effective in terms of calibration for these majority classes. This highlights the importance of using diverse and balanced metrics when evaluating model performance.

# Supplementary References

# References

1. Huang, G., Liu, Z., van der Maaten, L. & Weinberger, K. Q. Densely Connected Convolutional Networks. Preprint at https://arxiv.org/abs/1608.06993 (2016).

2. Zhou, Z., Siddiquee, M. M. R., Tajbakhsh, N. & Liang, J. UNet++: A Nested U-Net Architecture for Medical Image Segmentation. *Deep Learning in Medical Image Analysis and Multimodal Learning for Clinical Decision Support*, Lecture Notes in Computer Science, 3-11, <https://doi.org/10.1007/978-3-030-00889-5_1>, Springer International Publishing, Cham. (2018).

3. Chen, L.-C., Zhu, Y., Papandreou, G., Schroff, F. & Adam, H. Encoder-Decoder with Atrous Separable Convolution for Semantic Image Segmentation. *Proceedings of the European Conference on Computer Vision (ECOV)*, Springer, Cham. (2018).

4. Long, J., Shelhamer, E. & Darrell, T. Fully Convolutional Networks for Semantic Segmentation. *Proceedings of the IEEE Conference on Computer Vision and Pattern Recognition (CVPR)* 3431 – 3400 (2015).

5. Tan, M. & Le, Q. V. EfficientNet: Rethinking model scaling for convolutional Neural Networks. In *International Conference on Machine Learning.* **97**, 6105-6114 (2019).

6. Gut, D., Tabor, Z., Szymkowski, M., Rozynek, M., Kucybala, I. & Wojciechowski, W. Benchmarking of Deep Architectures for Segmentation of Medical Images. *IEEE Transactions on Medical Imaging.* **41**, 3231-3241 (2022).

7. Isensee, F., Jaeger, P. F., Kohl, S. A. A., Petersen, J. & Maier-Hein, K. H. nnU-Net: a self-configuring method for deep learning-based biomedical image segmentation. [*Nat. Methods* **18**, 203–211 (2021).](http://paperpile.com/b/KpWYi9/8Uxtz)

8. [Huo, X. *et al.*](http://paperpile.com/b/KpWYi9/YpBGg) Comprehensive AI model development for Gleason grading: From scanning, cloud-based annotation to pathologist-AI interaction. [*SSRN Electron. J.*](http://paperpile.com/b/KpWYi9/YpBGg) (2022)

9. [Koziarski, M. *et al.* DiagSet: a dataset for prostate cancer histopathological image classification. *Sci Rep* **14**, 6780 (2024).](http://paperpile.com/b/KpWYi9/VRlXo)

10. [Höhn, J. *et al.* Colorectal cancer risk stratification on histological slides based on survival curves predicted by deep learning. *NPJ Precis Oncol* **7**, 98 (2023).](http://paperpile.com/b/KpWYi9/ZUUnV)

11. [Geirhos, R. *et al.* Shortcut learning in deep neural networks. *Nature Machine Intelligence* **2**, 665–673 (2020).](http://paperpile.com/b/KpWYi9/YtbEW)

12. Pearson, K. X. On the criterion that a given system of deviations from the probable in the case of a correlated system of variables is such that it can be reasonably supposed to have arisen from random sampling. [*Lond. Edinb. Dublin Philos. Mag. J. Sci.* **50**, 157–175 (1900).](http://paperpile.com/b/KpWYi9/lkzG)
