# Supplementary material for: Pathologist-like explainable AI for interpretable Gleason grading in prostate cancer
Source: Nat Commun. 2025 Oct 8;16:8959. doi: 10.1038/s41467-025-64712-4 (PMC12508442; doi:10.1038/s41467-025-64712-4)
Supplement: Supplementary file 2 — Reporting Summary [file 41467_2025_64712_MOESM2_ESM.pdf]

nature portfolio

Corresponding author(s): Titus J. Brinker

Last updated by author(s): Aug 20, 2025

Reporting Summary

Nature Portfolio wishes to improve the reproducibility of the work that we publish. This form provides structure for consistency and transparency in reporting. For further information on Nature Portfolio policies, see our Editorial Policies and the Editorial Policy Checklist.

Statistics

For all statistical analyses, confirm that the following items are present in the figure legend, table legend, main text, or Methods section.

n/a	Confirmed	
☐	☒	The exact sample size (*n*) for each experimental group/condition, given as a discrete number and unit of measurement
☐	☒	A statement on whether measurements were taken from distinct samples or whether the same sample was measured repeatedly
☒	☐	The statistical test(s) used AND whether they are one- or two-sided<br>*Only common tests should be described solely by name; describe more complex techniques in the Methods section.*
☒	☐	A description of all covariates tested
☒	☐	A description of any assumptions or corrections, such as tests of normality and adjustment for multiple comparisons
☐	☒	A full description of the statistical parameters including central tendency (e.g. means) or other basic estimates (e.g. regression coefficient) AND variation (e.g. standard deviation) or associated estimates of uncertainty (e.g. confidence intervals)
☒	☐	For null hypothesis testing, the test statistic (e.g. *F*, *t*, *r*) with confidence intervals, effect sizes, degrees of freedom and *P* value noted<br>*Give P values as exact values whenever suitable.*
☒	☐	For Bayesian analysis, information on the choice of priors and Markov chain Monte Carlo settings
☒	☐	For hierarchical and complex designs, identification of the appropriate level for tests and full reporting of outcomes
☒	☐	Estimates of effect sizes (e.g. Cohen's *d*, Pearson's *r*), indicating how they were calculated

Our web collection on statistics for biologists contains articles on many of the points above.

Software and code

Policy information about availability of computer code

Data collection	Annotations were created and gathered with PlainSight (https://plainsight.ai).
Data analysis	All code was written in Python (3.10.13). PyTorch (2.1.1), PyTorch Lightning (2.2.0.post0), Albumentations (1.3.1), Pillow (9.5.0), Openslide (1.4.2), Pyvips (3.0.0), Shapely (2.1.1), OpenCV (4.8.1.78), MONAI (1.3.0), Hydra (1.3.2), NumPy (2.2.6), Pandas (2.1.1), Timm (0.9.2), WandB (0.17.1), Tensorboard (2.16.2), Omegaconf (2.3.0), SciPy (1.11.3), Scikit-learn (1.3.2), Scikit-image (0.22.0), Statsmodels (0.13.2), Matplotlib (3.8.0), and Seaborn (0.13.2) were used for image processing, model development and training, data analysis, and visualisation. The code for the analysis and the training of the model is available at https://github.com/DBO-DKFZ/GleasonXAI.

For manuscripts utilizing custom algorithms or software that are central to the research but not yet described in published literature, software must be made available to editors and reviewers. We strongly encourage code deposition in a community repository (e.g. GitHub). See the Nature Portfolio guidelines for submitting code & software for further information.

Data

Policy information about availability of data

All manuscripts must include a data availability statement. This statement should provide the following information, where applicable:

- Accession codes, unique identifiers, or web links for publicly available datasets
- A description of any restrictions on data availability
- For clinical datasets or third party data, please ensure that the statement adheres to our policy

The annotation data based on all three datasets generated in this study and the TMA core images of the TissueArray.com LLC dataset used in this study have been

deposited in the Figshare repository at https://doi.org/10.6084/m9.figshare.27301845 65.
The TMA core images of the Gleason 19 challenge used in this study are available on the Grand Challenge platform at https://gleason2019.grand-challenge.org/Register/.
The TMA core images of the Arvaniti et al. Harvard Dataverse dataset used in this study are available in the Harvard Dataverse repository at https://doi.org/10.7910/DVN/OCYCMP.
The WSI images of the AGGC dataset used in this study in the Supplementary Notes are available on the Grand Challenge platform. Access can be obtained after registration at https://aggc22.grand-challenge.org.
The WSI images of the DiagSet dataset used in this study in the Supplementary Notes can be obtained by registering at the database and providing a description of the intended use at https://ai-econsilio.diag.pl.
Source data are provided with this paper.

Research involving human participants, their data, or biological material

Policy information about studies with human participants or human data. See also policy information about sex, gender (identity/presentation), and sexual orientation and race, ethnicity and racism.

Reporting on sex and gender	We did not take gender or sex into consideration during the selection of the TMA core images.<br>We did not collect any data on sex and gender of the pathologists participating in our study.
Reporting on race, ethnicity, or other socially relevant groupings	No such categorization variables were used in our study.
Population characteristics	All included TMA core images contain prostate adenocarcinoma tissue with Gleason Patterns 3, 4, and 5. Further clinical data such as age, genotypic information and further diagnosis was not available for all datasets and not taken into account.<br>The pathologists in the reader study had a median of 15 years of clinical experience in pathology, with individual experience ranging from one to 35 years.
Recruitment	Pathologists were recruited from university clinics, non-university public clinics, and private pathology practices through the ISUP platform or direct email invitations.
Ethics oversight	The research complies with all ethics regulations. The study's ethics vote was approved by the ethics committee of the University Clinic Mannheim of the Medical Faculty of the University of Heidelberg, since our research involved no patients and no patient data was collected. Informed consent was obtained from all participating pathologists who performed the annotations of the publicly available data sets.

Note that full information on the approval of the study protocol must also be provided in the manuscript.

Field-specific reporting

Please select the one below that is the best fit for your research. If you are not sure, read the appropriate sections before making your selection.

- [x] Life sciences
- [ ] Behavioural & social sciences
- [ ] Ecological, evolutionary & environmental sciences

For a reference copy of the document with all sections, see nature.com/documents/nr-reporting-summary-flat.pdf

Life sciences study design

All studies must disclose on these points even when the disclosure is negative.

Sample size	The sample size was not predetermined using statistical methods. Two publicly accessible datasets were selected and expanded by a purchased dataset, thereby increasing the number of diverse sources and images. Successful models were developed with the public data in the field of Gleason Scoring, thereby providing reasonable grounds to assume that the combination of the datasets is sufficient for training on this more challenging task of explanation prediction.<br>Each TMA core image used in the development was annotated by three pathologists, which exceeds the usual standard of care in Gleason grading and allows for an estimation of uncertainty between the pathologists. The number of annotators per image was limited due to the detailed nature of the task and the associated effort of the pathologists as well as time constraints.
Data exclusions	Prior to the explanation annotation, images displaying Gleason Pattern 1 or 2, or those of benign tissue or different tumor tissue were excluded. If issues arose during the merging of the Gleason pattern annotation, such as too few annotators or no overlap, the images in question were excluded after review. Following the completion of the explanation annotations, images for which annotations had not been received from three pathologists by the end of the process were removed. This occurred primarily due to late dropouts of annotators, but also due to single images being skipped. A review of the images that had been skipped was conducted to ascertain whether any systematic biases had been introduced; however, no consistent issues were identified.
Replication	The performance of the AI was found to be consistent across different network initialisations and training data iteration orders. Replication of identical results is feasible through the utilisation of fixed seeds for the random generator during the training phase.<br>Each TMA core image was annotated by three annotators. The annotation showed a high amount of interrater variability.
Randomization	The data was split into training, validation and test data. The division was conducted in such a way as to minimise the L1-norm between the class distributions of the pixels between the splits, thereby ensuring the availability of each class in each split.<br>The annotators were grouped to ensure a mean experience of at least 10 years within groups, except in one group, where the average experience is slightly below 10 years due to a unforeseen dropout of a senior pathologist. The group of annotators were randomly assigned a

distinct set of TMA core images.

Blinding	The test set containing 15% of the data was withheld from the training and tuning of the model.<br>The annotators were blinded to the annotations of the other pathologists.

Reporting for specific materials, systems and methods

We require information from authors about some types of materials, experimental systems and methods used in many studies. Here, indicate whether each material, system or method listed is relevant to your study. If you are not sure if a list item applies to your research, read the appropriate section before selecting a response.

Materials & experimental systems

n/a	Involved in the study
☒	☐ Antibodies
☒	☐ Eukaryotic cell lines
☒	☐ Palaeontology and archaeology
☒	☐ Animals and other organisms
☒	☐ Clinical data
☒	☐ Dual use research of concern
☒	☐ Plants

Methods

n/a	Involved in the study
☒	☐ ChIP-seq
☒	☐ Flow cytometry
☒	☐ MRI-based neuroimaging

Plants

Seed stocks	*Report on the source of all seed stocks or other plant material used. If applicable, state the seed stock centre and catalogue number. If plant specimens were collected from the field, describe the collection location, date and sampling procedures.*
Novel plant genotypes	*Describe the methods by which all novel plant genotypes were produced. This includes those generated by transgenic approaches, gene editing, chemical/radiation-based mutagenesis and hybridization. For transgenic lines, describe the transformation method, the number of independent lines analyzed and the generation upon which experiments were performed. For gene-edited lines, describe the editor used, the endogenous sequence targeted for editing, the targeting guide RNA sequence (if applicable) and how the editor was applied.*
Authentication	*Describe any authentication procedures for each seed stock used or novel genotype generated. Describe any experiments used to assess the effect of a mutation and, where applicable, how potential secondary effects (e.g. second site T-DNA insertions, mosiacism, off-target gene editing) were examined.*
